# LEGO-Lipophosphonoxin membrane activity is enhanced by presence of phosphatidylethanolamine but hindered by outer membrane

**DOI:** 10.1038/s41598-024-83205-w

**Published:** 2025-01-07

**Authors:** Hana Brzobohatá, Milica Dugić, Viktor Mojr, Nitjawan Sahatsapan, Ivana Kóšiová, Tomáš Křížek, Tereza Dolejšová, Petra Lišková, Lukasz Cwiklik, Dominik Rejman, Radovan Fišer, Gabriela Mikušová

**Affiliations:** 1 Department of Genetics and Microbiology, Faculty of Science, Charles University, Viničná 5, 128 00 Prague, Czech Republic; 2 Institute of Organic Chemistry and Biochemistry, Czech Academy of Sciences v.v.i., Flemingovo náměstí 2, 166 10 Prague 6, Czech Republic; 3 Department of Analytical Chemistry, Faculty of Science, Charles University, Hlavova 8, 128 00 Prague, Czech Republic; 4 J. Heyrovský Institute of Physical Chemistry, Czech Academy of Sciences v.v.i., Dolejškova 3, 182 23 Prague, Czech Republic

**Keywords:** Antimicrobials, Medicinal chemistry

## Abstract

Finding effective antibiotics against multi-resistant strains of bacteria has been a challenging race. Linker-Evolved-Group-Optimized-Lipophosphonoxins (LEGO-LPPOs) are small modular synthetic antibacterial compounds targeting the cytoplasmic membrane. Here we focused on understanding the reasons for the variable efficacy of selected LEGO-LPPOs (**LEGO-1**, **LEGO-2**, **LEGO-3**, and **LEGO-4**) differing in hydrophobic and linker module structure and length. **LEGO-1–4** permeabilized cytoplasmic membrane of *Staphylococcus aureus*, *Bacillus subtilis*, *Pseudomonas aeruginosa*, and *Escherichia coli*, **LEGO-1** with the longest linker module being the most effective. Gram-positive bacteria were more sensitive to LEGO-LPPO action compared to Gram-negatives, which was manifested as a delayed membrane permeabilization, higher minimal inhibitory concentration and lower amount of LEGO-LPPO bound to the cells. Outer membrane permeability measurements and time-kill assay showed that presence of the intact outer membrane brought about reduced susceptibility of Gram-negatives. Using liposome leakage and in silico simulations, we showed that membranes with major content of phosphatidylethanolamine were more prone to LEGO-LPPO permeabilization. The proposed mechanism stems from an electrostatic repulsion between highly positively charged **LEGO-1** molecules and positively charged amino groups of phosphatidylethanolamine which destabilizes the membrane. Collectively, these data suggest that LEGO-LPPO membrane activity is enhanced by presence of phosphatidylethanolamine but hindered by presence of intact outer membrane.

## Introduction

Bacterial resistance to conventional antibiotics used in clinical practice is one of the major global issues of our era. It is estimated that in the future the anticipated death toll caused by drug-resistant infections may be compared with the global fatality rate of the current SARS-CoV-2 (COVID-19) pandemic^1^. Thus, there is a need not only to find mechanisms of resistance and the processes that microorganisms use to adapt to antibiotics but most importantly to find and test new active substances and thoroughly understand their mechanisms of action. In strong contrast, only about 30–40 new antibacterial compounds are currently in the clinical trial phases and the development of new antimicrobials is often based on repurposing existing drugs or using derivatives of established compound classes. Strikingly, this applies also to candidate compounds targeting World Health Organization priority pathogens that are only derivatives of existing antibiotic classes^2^.

For decades natural antimicrobial peptides (AMPs) have received substantial attention as potential alternatives to traditional antibiotics due to their broad-spectrum antimicrobial properties and specific mode of action and thus lower propensity to resistance development. Unfortunately, AMPs’ therapeutic development has been limited by their high production cost, low biological activity, susceptibility to proteases, and cytotoxicity^3^. Need for new antimicrobial therapeutics potentiated several other alternative antibiotic approaches. These include not only synthetic AMPs, but also antisense therapeutics which can target and silence any bacterial gene. Other include antibodies and antibody–antibiotic conjugates, lytic bacteriophages or engineered phages inhibiting a range of targets by delivery of CRISPR–Cas, and microbiota-based therapies^4^. With the development of AI technology combined with high-throughput screening techniques diverse libraries of compounds can be screened and antimicrobial and hemolytic properties can be predicted^5,6^.

Another valuable source of new antimicrobials are amphiphilic small molecule antimicrobials^7^ such as small synthetic compounds. Such compounds seem to be promising because of their relative simplicity and thus easy and cost-effective production. Further, chemical synthesis offers to use also nonproteinaceous building blocks, to obtain high yields and to fine-tune their antimicrobial potential via subtle changes in the structure which might bring about changes in activity.

Among the possible target sites of antimicrobial agents, the cytoplasmic membrane is yet not well exploited^8,9^. The cytoplasmic membrane is essential to bacterial cell as it is its only membrane structure where numerous membrane-based processes take place. To be able to perform both its barrier function and provide a stable environment for vital processes of the cell, the membrane must be relatively complexly organized and actively maintained in an optimal state^10^. Thus, it can be assumed that unlike a particular enzyme or signaling pathway a simple point mutation cannot provide resistance to a membrane-damaging agent while maintaining the proper function of membrane-bound cellular processes at the same time. Several substances are known to disrupt membrane barrier function, ranging from antimicrobial peptides, nonribosomally produced molecules to synthetic antimicrobial peptidomimetics^11,12^.

Several years ago, we developed promising antibacterial compounds termed lipophosphonoxins (LPPOs) exhibiting significant antibacterial activities against a wide range of bacteria, including multidrug-resistant strains, with no cytotoxicity on human cells at bactericidal concentrations^13–15^. LPPOs act through permeabilization of the bacterial membrane leading to its disruption and cell death. So far, we have synthesized and tested three generations of LPPOs. The first LPPO generation^16^ displayed significant antibacterial activities against Gram-positive pathogens. Further modifications led to the second LPPO generation with improved antibacterial activities against both Gram-positive and Gram-negative bacteria^15^. Consequently, selected second generation LPPOs have been successfully evaluated as additives to surgical bone cements to prevent infections^17^ and as component of nanofiber dressing capable of reducing wound infection in mice^18^.

**Fig. 1:**
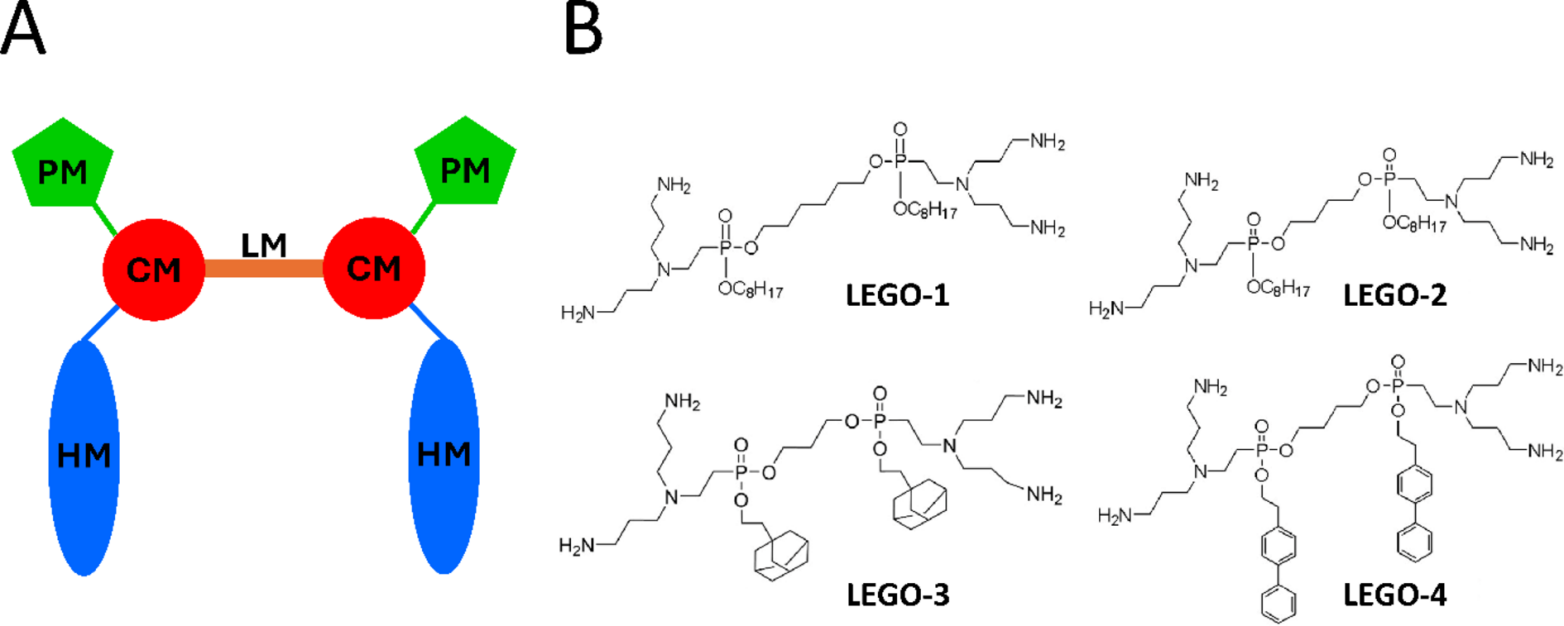
(**A**) General structure of LEGO-LPPOs containing polar modules (PM), connector modules (CM), hydrophobic modules (HM), and a linker module (LM). (**B**) Chemical structure of selected LEGO-LPPOs molecules used in this study.

Further structure optimization of second generation LPPO failed to overcome the inhibition of their antimicrobial action by the presence of serum albumins. Thus, inspired by symmetrical peptidomimetics^19,20^ we designed new modular structure loosely based on LPPO, termed Linker-Evolved-Group-Optimized-Lipophosphonoxins (LEGO-LPPO)^18^. LEGO-LPPOs consist of two polar modules (PM), two connector modules (CM), two hydrophobic modules (HM) and one linker module (LM) as depicted in Fig. 1A.

In the previous paper^18^ we synthesized a large set (several dozens) of LEGO-LPPO molecules and characterized them by antimicrobial activity, mode of action, and cytotoxicity (Suppl. Table 1). In the current study, we present a detailed study of four selected LEGO-LPPOs from this set. We selected molecule **38** which is designated as **LEGO-1** here, molecule **25** which is designated as **LEGO-2** here, and molecule **23** which is designated as **LEGO-3** here (Fig. 1B, Table 1). Additionally, we have synthesized **LEGO-4** which is not only highly active in terms of minimum inhibitory concentration (MIC) values but at the same time it possesses fluorescent properties, which enabled us to detect the LEGO-LPPO interaction with target membrane. All selected compounds are composed of the same polar module: dipropylentriamine moiety attached to the ethylphosphonate connector module. **LEGO-1**, one of the most potent LEGO-LPPOs^18^ in this series holds six carbon atoms long linker and C8 linear alkyl chains as HM. To test the effect of shorter LM, **LEGO-2** was selected—it has four carbon atoms long linker and identical HM as **LEGO-1**. Other two molecules were included to test how the nature of HM affects LEGO-LPPO activity: **LEGO-3** and **LEGO-4**, having C3 and C4 long LM, respectively, and adamantylethyl and aromatic biphenylethyl as HM, respectively.

**Table 1:** Structural and physico-chemical characteristics of selected LEGO-LPPOs molecules used in this study.

Compound	PM	LM	HM	*M*_*r*_ (g/mol)	*CHIg*	*cLogD* (pH 7.2)	*CMC* (mM)
LEGO-1		6 C	Aliphatic	1003.8	36.9	1.81	1.42
LEGO-2		4 C	Aliphatic	975.8	35.3	1.38	1.32
LEGO-3		3 C		1061.9	36.9	2.42	1.89
LEGO-4		4 C		1111.9	32.8	3.12	1.49

## Results

### Antibacterial activities of selected LEGO-LPPOs

We compared antibacterial activity of **LEGO-1–4** (Fig. 1) differing in the length of linker module and/or structure of hydrophobic modules (Table 1). MIC were assessed for Gram-positive bacterial strains of *Staphylococcus aureus* CCM 4223 and *Bacillus subtilis* 168 and for Gram-negative bacterial strains of *Pseudomonas aeruginosa* CCM 3955 and *Escherichia coli* CCM 3954. *Escherichia coli* imp4213 with a compromised outer membrane was also involved to assess the role of intact outer membrane in sensitivity to LEGO-LPPOs. This strain bears an in-frame deletion of the *lptD* gene encoding the essential outer membrane protein LptD, which is involved in LPS assembly^21^, and thus produces permeability defects of the outer membrane.

Generally, Gram-positive bacteria and *E. coli* imp4213 with compromised outer membrane were more sensitive to LEGO-LPPO action. This applies to the whole large set of dozens LEGO-LPPOs in general, as shown in Suppl. Fig. 1 which compares MIC of all previously published LEGO-LPPOs against tested Gram-positive and Gram-negative bacterial strains. The results in Table 2 show that despite the differences in the chemical structure all the tested LEGO-LPPOs molecules exert potent antimicrobial activity which is comparable in terms of MIC. The longer LM of **LEGO-1** (C6) promotes antimicrobial activity in comparison to shorter (C3 or C4) linkers of **LEGO-2**, **LEGO-3** and **LEGO-4** against tested Gram-negative strains. Activity against *S. aureus, B. subtilis* and *E. coli* imp4213 is comparable. Substitution of the aliphatic group of **LEGO-2** by a biphenyl residue in the HM in **LEGO-4** slightly lowers MIC against *B. subtilis* and *S. aureus*, whereas the MIC against *P. aeruginosa* and both *E. coli* strains remains unchanged or is even higher. Compared to **LEGO-2**, presence of adamantylethyl moieties as HM of **LEGO-3** increases activity against *S. aureus* and *B. subtilis*, however substantially decreases activity against *P. aeruginosa* and *E. coli* strains.

**Table 2:** Antimicrobial (MIC, mg/L) and hemolytic (HC_50_) activity of selected LEGO-LPPOs.

Compound	*E. coli* CCM 3954	*P. aeruginosa* CCM 3955	*S. aureus* CCM 4223	*B. subtilis* 168	*E. coli* imp4213	HC_50_ (mg/L)
LEGO-1	2	4	2	1	1	377
LEGO-2	4	16	8	2	1	> 2000
LEGO-3	4	8	2	1	0.5	313
LEGO-4	8	16	2	1	4	> 2000

Regarding the hemolytic activity of LEGO-LPPOs, **LEGO-1** with the longest LM is not only the most active against bacteria, but together with **LEGO-3** bearing the adamanthylethyl as HM generally the most penetrating one, including erythrocytes. Still their HC_50_ is two orders of magnitude higher than MIC.

### Inoculum effect of LEGO-LPPOs

**Fig. 2:**
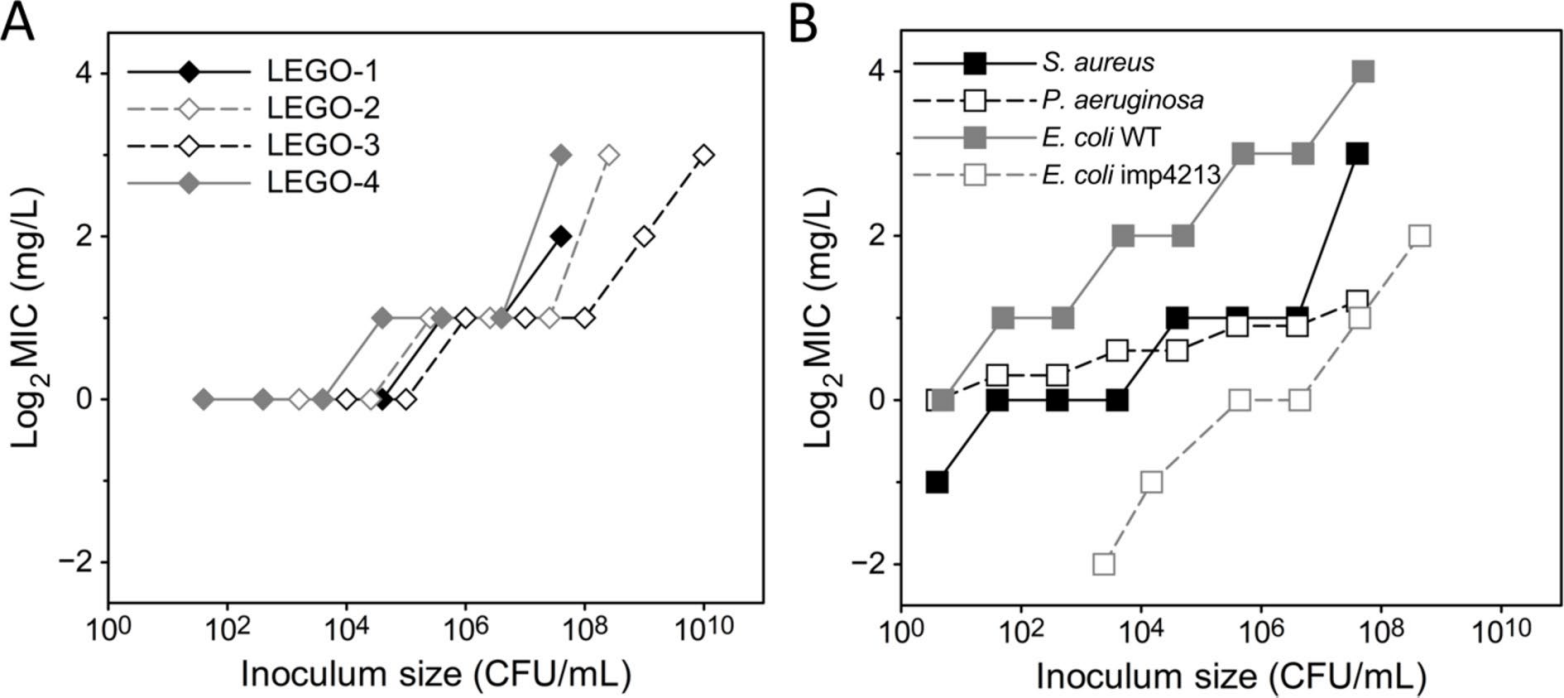
(**A**) Comparison of inoculum effect (IE) of **LEGO-1**, **-2**, **-3**, and **-4** on *Staphylococcus aureus.* The character of IE was similar for all four compounds but differed in the overall efficiency of LEGO-LPPOs against the tested bacterial strains. (**B**) Inoculum effect of **LEGO-4** on all tested bacterial strains shown as an increase in Log_2_(MIC) values, depending on inoculum size.

Inoculum effect (IE), also referred as collective bacterial tolerance, is a clinically relevant issue, that demonstrates as lower susceptibility (higher MIC) in higher bacterial cell densities^22,23^. As IE is an essential parameter of antibiotic efficiency, we wanted to investigate if it applies also for LEGO-LPPOs. We characterized the IE of LEGO-LPPOs as a change of MIC with increasing inoculum size ranging 10^3^–10^8^ CFU/mL after 24 h of incubation (Table 3, Fig. 2, Suppl. Figs. 2 and 3). IE observed for all four compounds had a similar character (Fig. 2A)—the tenfold increase in inoculum size resulted in almost doubling of the MIC (MIC increased 1.25–1.8 times, Table 3). Such a change in MIC is regarded as a moderate inoculum effect (Suppl. Fig. 2). The IE was most intense in the case of *E. coli* imp4213 treated with **LEGO-4** and *S. aureus* with **LEGO-2**. The least intense of IE was in the case of *P. aeruginosa* with **LEGO-1**.

**Table 3:** Evaluation of inoculum effect calculated as the average of the ratios of two consecutive MIC values for inoculums with CFU differing ten-times (data from Fig. 2B) of each LEGO-LPPOs concentration series. Significant IE has been defined as a ≥ 8-fold change in MIC when an inoculum 100-fold greater than the CLSI^24^ recommendation is used^25^.

	LEGO-1	LEGO-2	LEGO-3	LEGO-4
*S. aureus*	1.7	1.8	1.4	1.7
*P. aeruginosa*	1.25	1.7	1.7	1.6
*E. coli* WT	N/A	N/A	N/A	1.6
*E. coli* imp4213	N/A	N/A	N/A	1.8

### Mechanism of liposome leakage

Liposomes of size 1000 nm, filled with ANTS^+^/DPX^-^ solution were used to characterize leakage mechanism and anion/cation selectivity of pores formed by LEGO-LPPOs. Membrane active compounds can disrupt liposomes in two different ways—by an “all-or-none” mechanism or a graded leakage^26^. To test and compare the lytic activities of each LEGO-LPPO we treated the liposomes composed of synthetic phospholipids mixtures containing DOPG and DOPE in a ratio 2:1 (m/m) and 1:2 (m/m) with different concentrations of LEGO-LPPOs at constant incubation time (*t* = 90 min). All compounds induced leakage of ANTS and DPX from liposomes. The amount of ANTS and DPX outside was calculated according to Eq. (1).

**Fig. 3:**
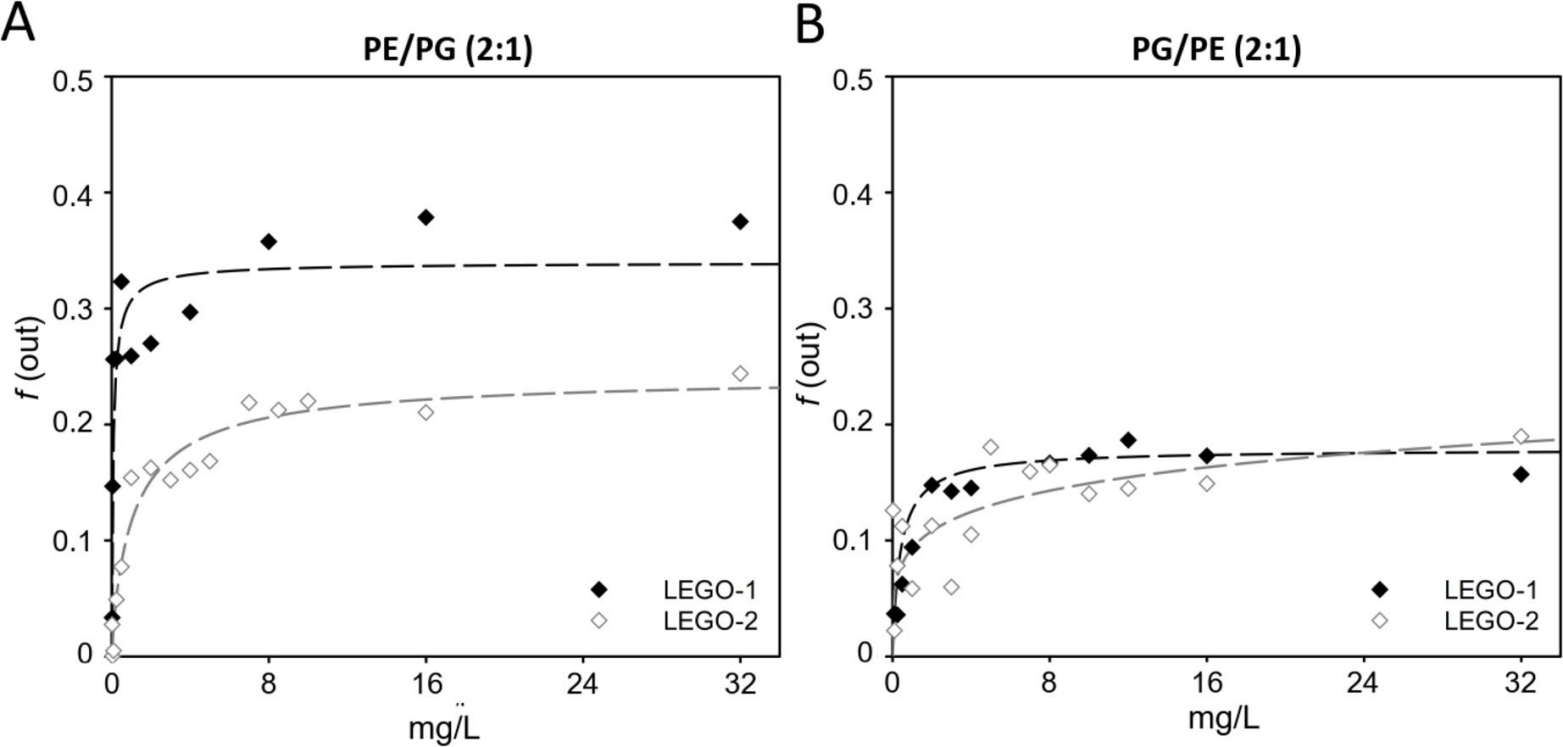
Concentration dependency of LEGO-LPPOs (**LEGO-1** and **LEGO-2**) permeabilization activity in PE/PG (**A**) and PG/PE (**B**) liposomes, shown as *f*_*out*_ (fraction of ANTS outside the liposomes). The results are fitted with the Hill function. Hill coefficients (Table 4) suggest no positive cooperativity of LEGO-LPPOs action on liposomes of both membrane compositions. Graphs show representative data. Results for **LEGO-3** and **LEGO-4** are presented in Suppl. Fig. 4.

The results (Fig. 3, Suppl. Fig. 4) show the fraction of ANTS outside the liposomes as a function of LEGO-LPPO concentration. The concentration dependency was then fitted with Hill function (Eq. 2, Table 4). The values of Hill coefficients (0.3 < *n*_*LEGO-LPPO*_ < 0.9) suggest no positive cooperativity in any LEGO-LPPO’s action on liposomes, surprisingly.

**Table 4:** Calculated Hill coefficients (*n*) of LEGO-LPPOs leakage curves presented in Fig. 3 and Suppl. Fig. 4.

Compound	PE/PG (2:1)	PG/PE (2:1)
LEGO-1	0.6	0.8
LEGO-2	0.9	0.3
LEGO-3	0.8	0.9
LEGO-4	0.6	0.8

**Fig. 4:**
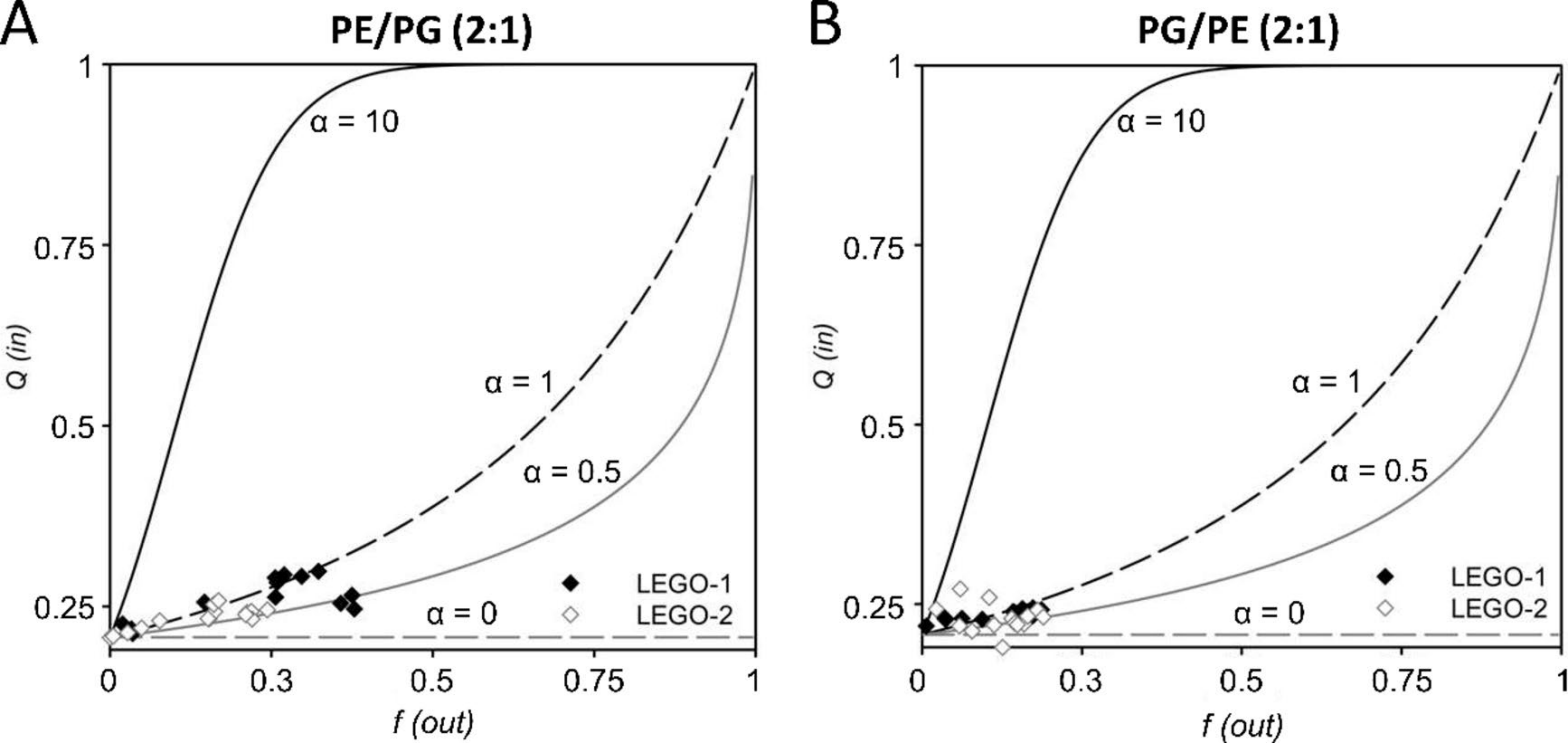
Results of requenching assay of **LEGO-1** and **LEGO-2** in PE/PG (**A**) and PG/PE (**B**) liposomes. Model α values (0, 0.5, 1 and 10) are shown. The estimated values $\alpha \in \langle 0.5;\; 1\rangle$ suggest that action mechanism could be graded, slightly anion selective leakage or a combination of all-or-none and graded non-selective leakage. See “Methods” for details of this assay.

We also tested the LEGO-LPPO membrane pores in terms of anion/cation selectivity based on the requenching method. All tested compounds induced leakage of fluorescent molecules from liposomes. Calculated values of *Q*_*in*_ were plotted as a function of *f*_*out*_ (Fig. 4, Suppl. Fig. 5) and α value was used to describe the action mechanism. Results suggest that despite the structural differences, the leakage mechanism is very similar for all four molecules. Parameter $\alpha \in \langle 0.5;\; 1\rangle$ (Table 5) implies that action mechanism could be graded, slightly anion selective leakage or a combination of all-or-none and gradual non-selective leakage. Although no significant difference in action mechanism was observed between the two phospholipid compositions, the leakage rate was generally higher on liposomes with major content of PE (i.e., PE/PG 2:1) in case of all four molecules.

**Table 5:** Calculated α values (Eq. 1) of LEGO-LPPOs curves presented in Fig. 4 and Suppl. Fig. 5.

Compound	PE/PG (2:1)	PG/PE (2:1)
LEGO-1	0.7	0.8
LEGO-2	0.7	0.6
LEGO-3	0.6	0.3
LEGO-4	0.6	0.4

### MD simulations of LEGO-1 in the membrane

We aimed at exploring at the molecular level what are the differences in the PG/PE (2:1) and PE/PG (2:1) membranes after treatment with LEGO-LPPO. We chose **LEGO-1** end employed atomistic MD simulations searching how frequent are specific **LEGO-1** contacts with phospholipids in these membranes, and if it could explain the higher **LEGO-1** activity in PE/PG (2:1).

**Fig. 5:**
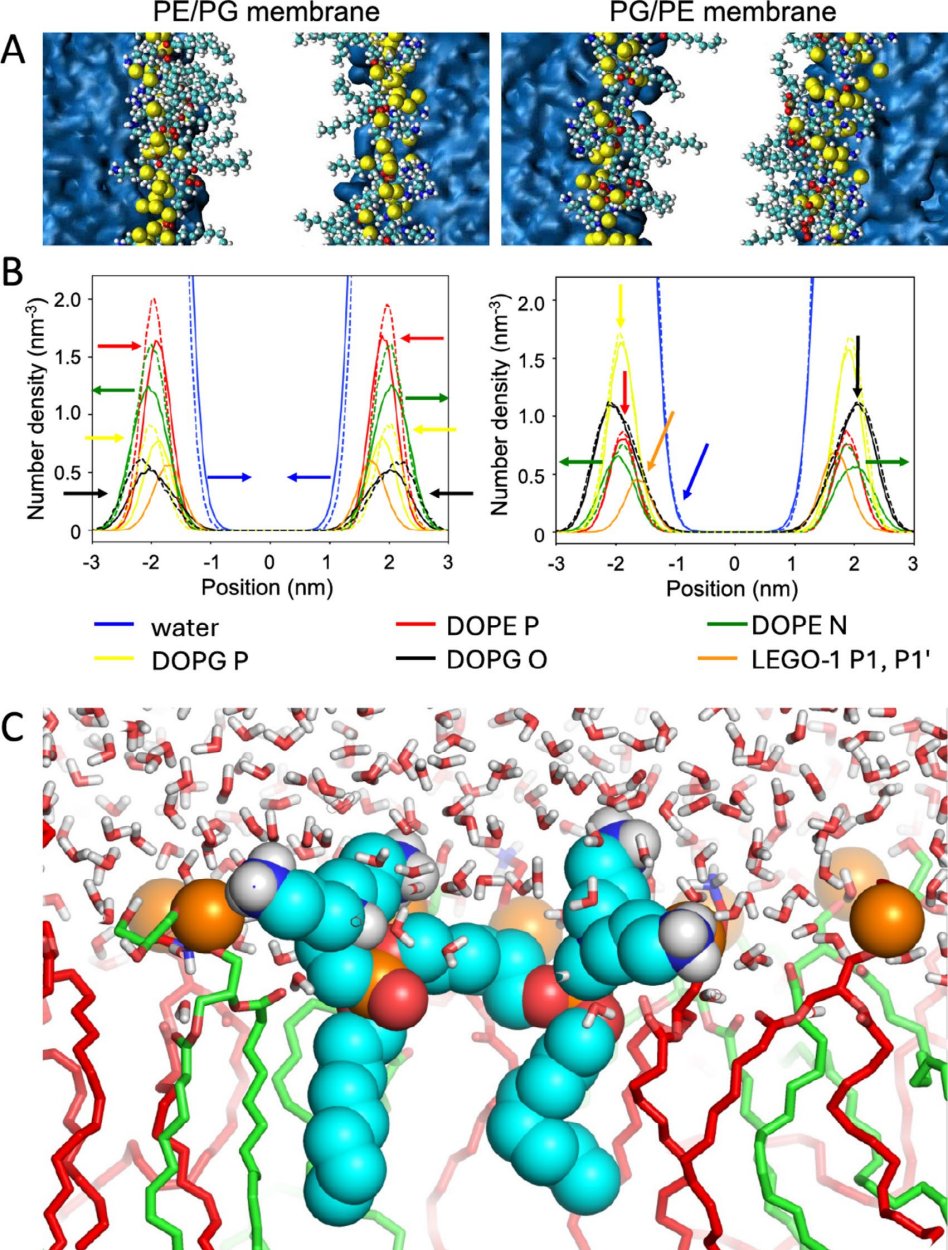
(**A**) Representative snapshots of membranes after 2000 ns of simulation of **LEGO-1** interaction with PE/PG and PG/PE membranes. (**B**) Density profiles showing the position of selected atoms (see legend). DOPE N is the amine group of ethanolamine, DOPG O are atoms OC2 and OC3 of the glycerol group. Dashed lines: profiles before **LEGO-1** addition into the system, solid lines: after **LEGO-1** addition. Note that phosphate groups of DOPE and DOPG (red and yellow arrows) together with glycerol group of DOPG (black) move deeper into the membrane after **LEGO-1** addition to PE/PG membrane (left) but not to PG/PE membrane (right). In the same time water molecules tend to penetrate the membrane after **LEGO-1** addition to PE/PG membrane (blue arrows). Red, yellow, and orange arrows highlight the depth of phosphate and phosphonate groups of DOPE, DOPG and **LEGO-1**, respectively. In PG/PE membrane **LEGO-1** induces extension of amino group of DOPE away from the membrane (right, green curves). (**C**) Example snapshot of **LEGO-1** molecule (cyan) buried inside the membrane (PE—red, PG—green) and allowing water to penetrate. Orange spheres indicate phosphate and phosphonate groups of phospholipids and **LEGO-1**, respectively. (**A**) and (**B**) were produced using VMD software and (**C**) using PyMOL software (for details see “Methods” section).

In both systems, the MD trajectory (2000 ns long, for details see “Methods”) can be split into two parts. The first ~ 500 ns is needed for **LEGO-1** molecules to adsorb and insert into the membrane. The rest of the trajectory was used for the analysis of the equilibrium state of the system. First, we studied the density profiles of the atoms across the membrane (Fig. 5). We observed that phosphonate groups of **LEGO-1** were buried about 0.2 nm deeper into the membrane in comparison to phosphate groups of both DOPE and DOPG (Fig. 5B—red, yellow, and orange arrows, 5C). At the same time, the amino group of DOPE extended away from the membrane after **LEGO-1** insertion (green arrows). Interestingly, selectively in PE/PG membrane, **LEGO-1** induced relocalization of glycerol groups and both DOPG and DOPE phosphate groups deeper into the membrane which was accompanied by penetration of water molecules into the membrane (Fig. 5B, black and blue arrows). The density profiles of P and N atoms in PE/PG membranes show decreased values after **LEGO-1** treatment which signifies less packed membrane that can expand laterally during the simulation (Fig. 5B, red and green curves).

**Fig. 6:**
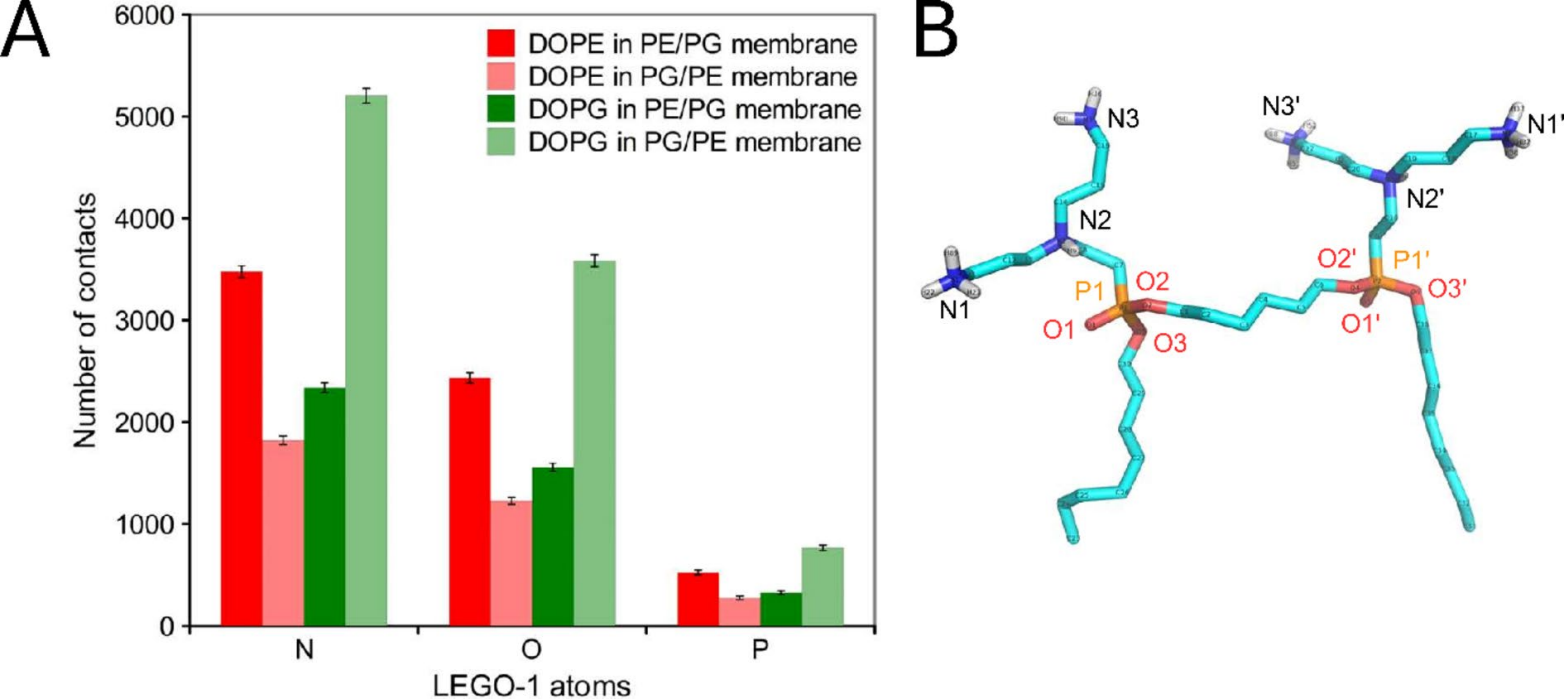
(**A**) Number of **LEGO-1** contacts with DOPE and DOPG atoms in both types of membranes (PE/PG and PG/PE), without any normalization. The error bars show the standard deviation estimated as square root of the observed counts (based on Poisson statistics). (**B**) **LEGO-1** molecule with numbered atoms.

In order to explain the observed changes in the membrane in more detail, we selected 100 snapshots from the interval 1000–2000 ns of MD simulation (separated by 10 ns) where we counted the atom–atom contacts at distance < 0.4 nm (only N, O and P atoms were taken into account, Fig. 6). Each **LEGO-1** molecule has about six to eight contacts in one moment, on average. Within this dataset, **LEGO-1** created 12,884 contacts in PG/PE membrane and 10,659 contacts in PE/PG membrane. Thus, the relative preference of LEGO-1 to PG/PE membranes is 1.21× (see Fig. 6). This observation is a bit surprising as in both systems the **LEGO-1** molecules are membrane-inserted according to visual inspection.

The increased number of **LEGO-1** contact in PG/PE membrane can be explained by highly preferred interaction with DOPG. In PG/PE membrane **LEGO-1** stays in contact with DOPG ~ 1.3× more frequently than expected from DOPG content in the PG/PE membrane. Similar effect was observed also in PE/PG membrane where **LEGO-1** was in contact with DOPG 1.18× more frequently than with DOPE when compared with theoretical stochastic distribution (according to the membrane composition and different number of DOPE, resp. DOPG polar atoms). **LEGO-1** binds to the membranes mostly by all primary amins (atoms N1/N1′ and N3/N3′) and tertiary amins (atoms N2/N2′). Surprisingly, oxygen atom O1 and O3 also play a substantial role in the interactions.

### Permeabilization of cytoplasmic membrane of living cells

We next tested the membrane permeabilization ability of LEGO-LPPOs in living cells using propidium iodide (PI) influx assay. In this assay we determined and compared the ability of each LEGO-LPPOs to perforate bacterial cytoplasmic membrane to such extent, that the PI molecules can enter the cells and bind to DNA followed by increase of fluorescence.

**Fig. 7:**
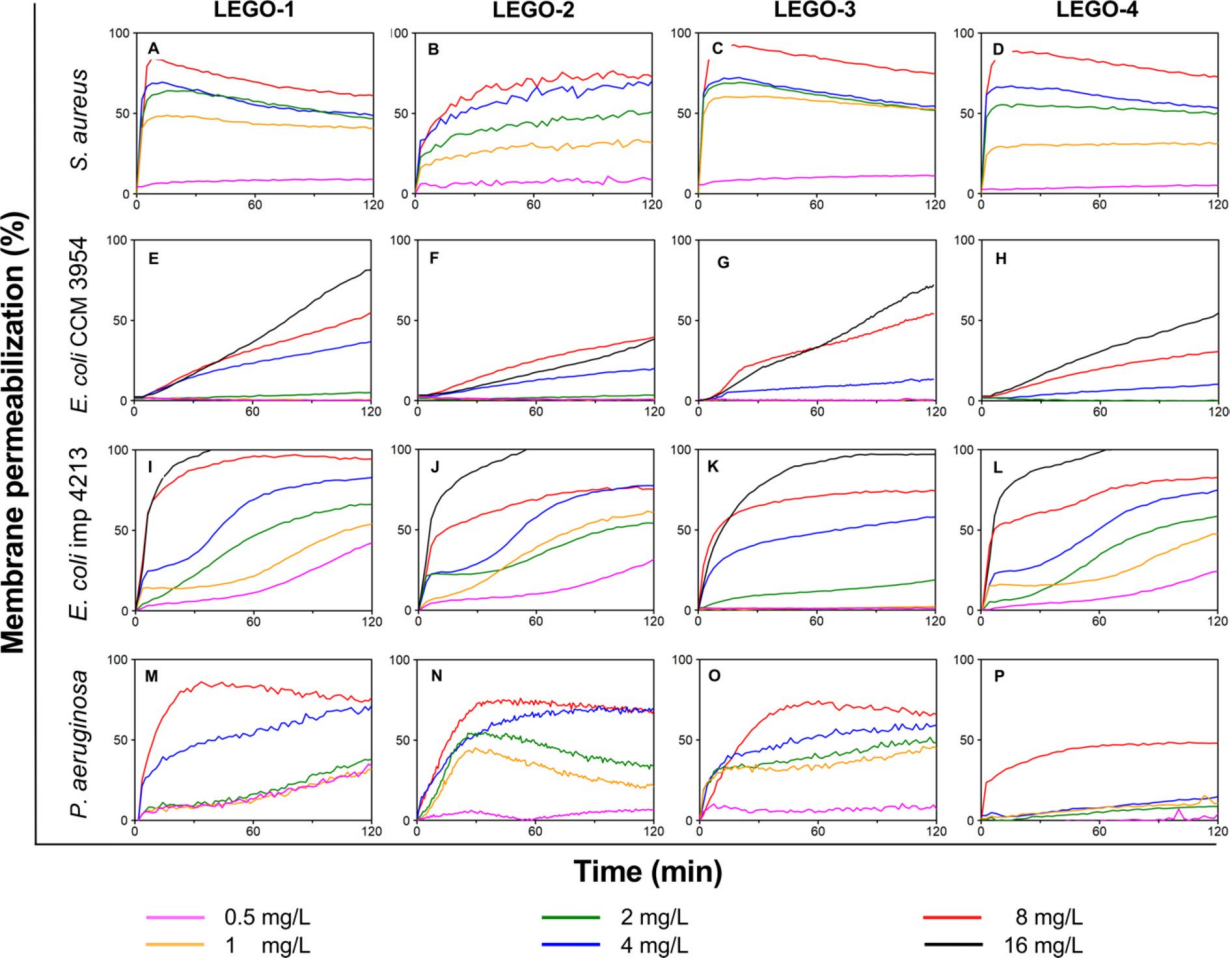
Permeabilization of cytoplasmic membrane of different bacteria induced by LEGO-LPPOs. Concentration range (0.5–16 mg/L). Representative curves are shown. Note that in some graphs the curves showing the lowest LEGO-LPPO concentrations used, which induced only a weak membrane permeabilization, overlap and thus are not visible.

We confirmed that all the tested LEGO-LPPOs possess perforating activity (Fig. 7). For *S. aureus*, *E. coli* imp4213 and *P. aeruginosa*, all LEGO-LPPOs induced very similar permeabilizing effect both in terms of the course of kinetic of lysis and maximum reached intensity. Exceptionally, *E. coli* CCM 3954 (Fig. 7E–H) was the least susceptible against all tested LEGO-LPPOs and **LEGO-4** had the lowest permeabilizing effect on *P. aeruginosa* (Fig. 7P)*.* These observations led us to conclude that chemical structure differences do not alter the kinetics of membrane permeabilization, concentration dependency nor LEGO-LPPO mechanism of action.

On the other hand, we noticed distinctive differences in kinetics of PI influx when comparing individual bacterial strains treated with the selected LEGO-LPPOs on comparable concentration range. Whereas using LEGO-LPPOs promoted rapid action in *S. aureus* (Fig. 7A–D) and in *E. coli* imp4213 (Fig. 7I–L), their permeabilizing potential was lower in *P. aeruginosa* (Fig. 7M–P). Interestingly, very slow onset of PI influx was observed in *E. coli* CCM 3954 and low final PI intensities were observed within the time interval of the experiment (Fig. 7E–H). When using **LEGO-4** in the concentration of 8 mg/L the rate of PI influx is more than ten-times higher in the case of *E. coli* imp4213 in the first 10 min of lysis (6.67%/min) compared to *E. coli* CCM 3954 (0.5%/min). In the case of *E. coli* imp4213 we observed fast action immediately after LEGO-LPPO addition, which is similar to the kinetics of membrane permeabilization of *S. aureus.* However, after 45 min of measurement, in *E. coli* imp4213 there appeared a secondary increase in fluorescence intensity. These two separated phases of action were not observable in any other bacterial strain, and it only appeared in some concentrations of **LEGO-1** (Fig. 7I, c = 1 and 4 mg/L), **LEGO-2** (Fig. 7J, c = 1, 2 and 4 mg/L) and **LEGO-4** (Fig. 7L, c = 1, 2, 4 and 8 mg/L).

**Fig. 8:**
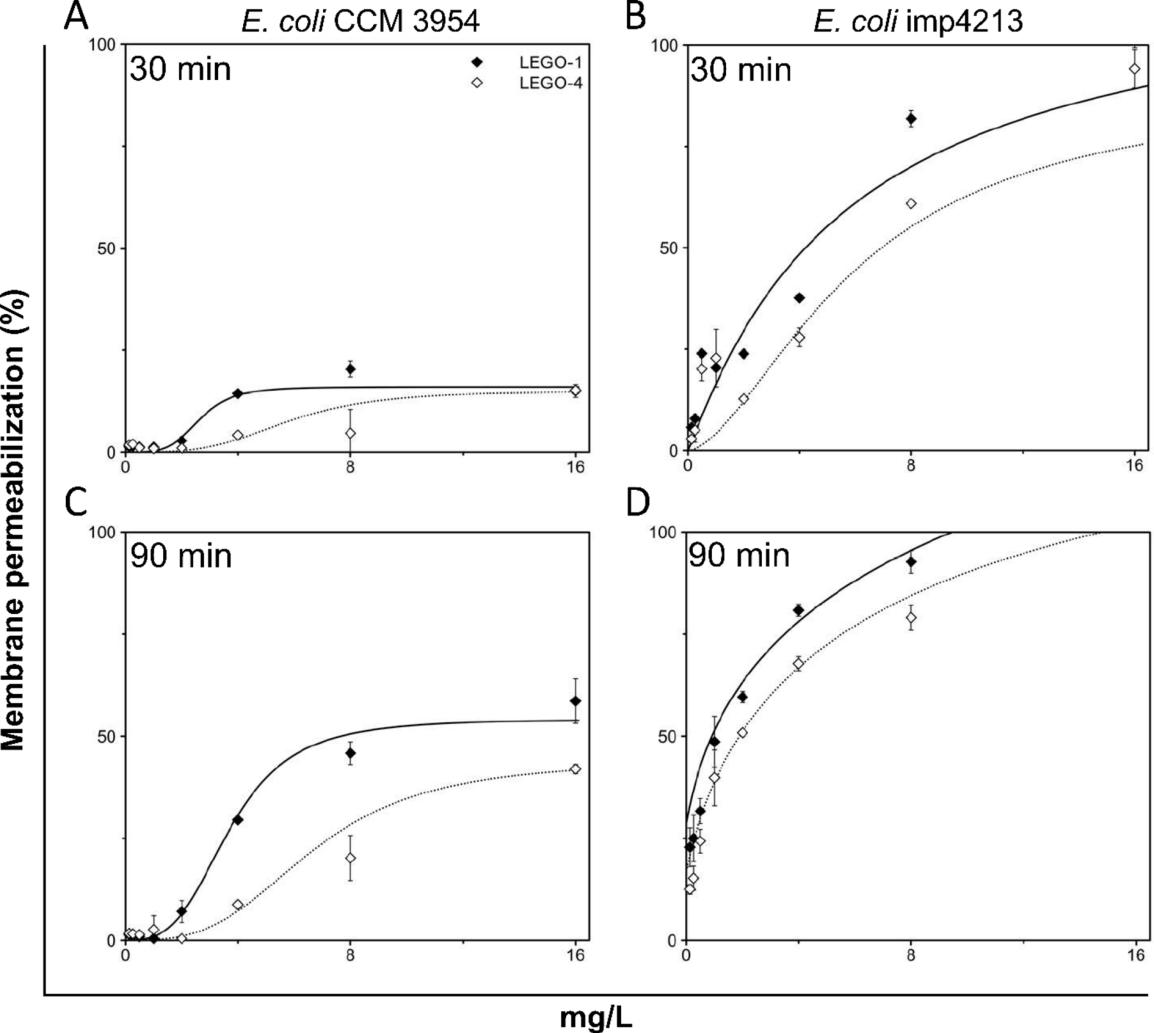
LEGO-LPPOs concentration dependency (**LEGO-1** and **LEGO-4** shown) of *E. coli* CCM 3954 (**A**,**C**) and *E. coli* imp4213 (**B**,**D**) cell permeabilization fitted with Hill function. The dependency is shown after 30 (**A**,**B**) and 90 min (**C**,**D**) of action. The presented data are averages (± SD) from at least two experiments performed in triplicate.

To better understand the differences in killing kinetics in *E. coli* imp4213 and *E. coli* CCM 3954, we compared the LEGO-LPPO concentration dependencies of membrane permeabilization in time of the primary (30 min) and the secondary (90 min) phase on both *E. coli* strains (Fig. 8, Suppl. Fig. 6). The sigmoidal dose–response curve of permeabilization supports the hypothesis of a primary and a secondary phase of action in *E. coli*. The LEGO-LPPOs concentration dependent activity was fitted with Hill function (Suppl. Tables 3 and 4). The calculated Hill coefficients suggest cooperative action in *E. coli* CCM 3954 at both phases (Fig. 8A,C) with *n*_*1*_ = 3.95 and *n*_*2*_ = 2.96 for **LEGO-1** and *n*_*1*_ = 2.53 and n_2_ = 2.36 for **LEGO-4** (at 30 and 90 min, respectively). On contrary, LEGO-LPPOs action on *E. coli* imp4213 (Fig. 8B,D) seems to be non-cooperative, with *n*_*1*_ = 0.91 and *n*_*2*_ = 0.83 for **LEGO-1** and *n*_*1*_ = 1.80 and *n*_*2*_ = 0.89 for **LEGO-4** (at 30 and 90 min, respectively). Thus, increased membrane permeability correlated with lower Hill number suggesting that the mechanism of action is different in the two *E. coli* strains. Considering the different shapes of concentration dependencies and dramatically different kinetics reached by LEGO-LPPOs in both *E. coli* strains, we hypothesized that the cell structure responsible for the possibly different mode of action is the outer membrane.

### Time-kill kinetics against *E. coli* CCM 3954 and *E. coli* imp4213

**Fig. 9:**
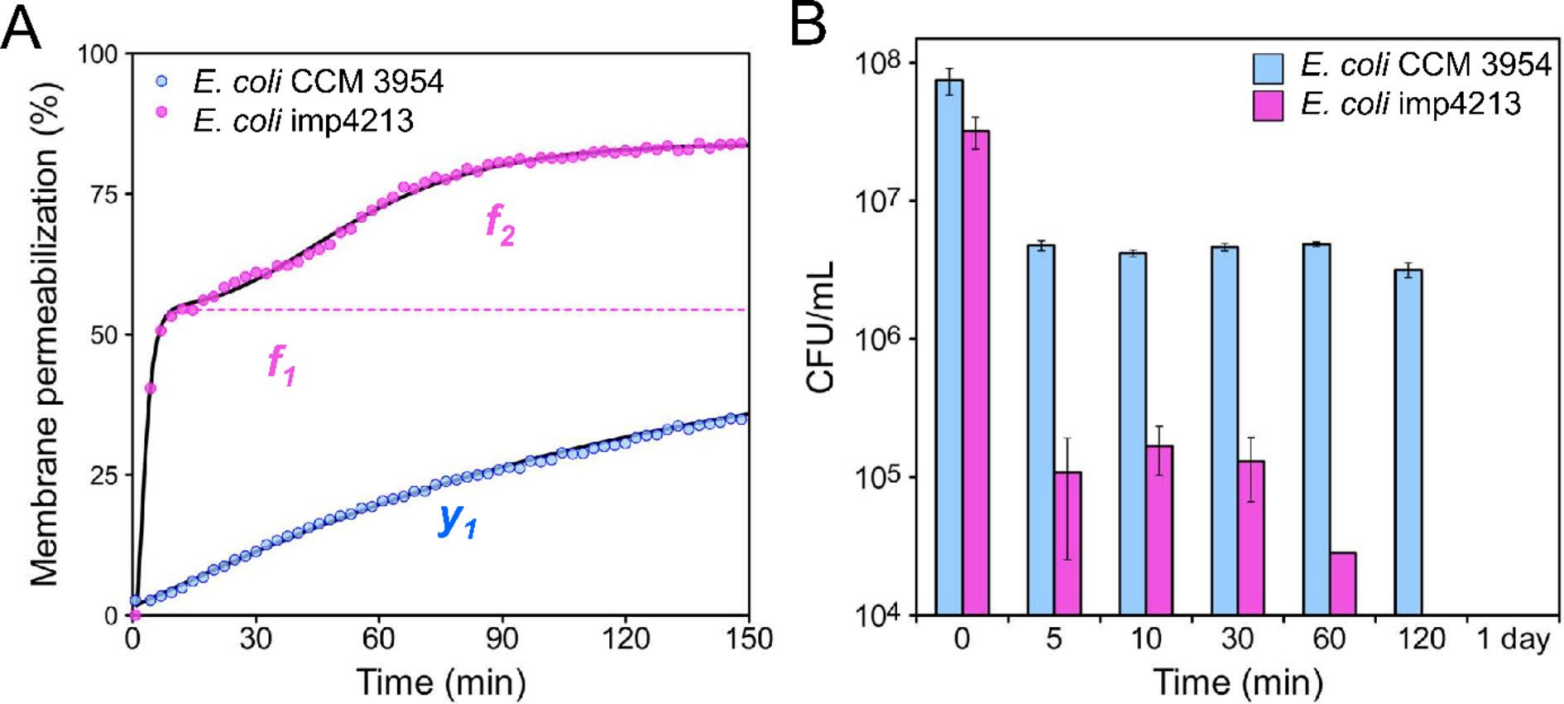
Time-kill experiments with **LEGO-4** against *E. coli* CCM 3954 and *E. coli* imp4213. (**A**) Membrane permeabilization of *E. coli* strains induced by 8 mg/mL **LEGO-4** (data from Fig. 7H,L, representative curves fitted with Hill function). The black lines show the fitted curve (Eq. 4). Two phases (*f*_*1*_ and *f*_*2*_) can be detected on *E. coli* imp4213 strain whereas single phase (*y*_*1*_) is observed on *E. coli* CCM 3954. (**B**) Effect of 8 mg/L of **LEGO-4** on cell viability at different time points indicated on *x* axes. The average CFU/mL ± SD is shown.

**LEGO-4** in the concentration of 8 mg/L showed the greatest difference in kinetics of PI entry between *E. coli* CCM 3954 and *E. coli* imp4213, respectively (Fig. 9A). Using this concentration, we evaluated and compared the effect of **LEGO-4** on cell viability in time. In case of both *E. coli* strains fast decrease in CFU/mL was observed within five minutes after **LEGO-4** exposure and the killing rate was steeper for *E. coli* imp4213 (Fig. 9A, f_1_ and Fig. 9B)—number of viable cells dropped down by three orders of magnitude. Additionally, we noticed a second decrease in cell counts during the time-kill assay, that corresponded to the second increase in fluorescence intensity (Fig. 9A, f_2_) that appeared after 60 min equally in PI and time-kill assay as well. In contrast, only the slow increase in PI fluorescence in *E. coli* CCM 3954 (Fig. 9A, *y*_*1*_) corresponded with an almost constant value of CFU/mL, which dropped down immediately after **LEGO-4** addition and remained at the same level within 120 min of measurement. After a 1-day incubation **LEGO-4** completely killed all bacteria of both strains. These results suggest that there might be two different action mechanisms of LEGO-LPPOs on *E. coli* membrane, one of which is blocked by the intact outer membrane in *E. coli* CCM 3954.

### Affinity of LEGO-LPPOs to bacterial cells

To better understand the different sensitivity and action mechanism of selected LEGO-LPPOs on Gram-positive and Gram-negative bacterial strains, we compared binding ability of **LEGO-4** to *S. aureus*, *P. aeruginosa*, *E. coli* CCM 3954 and *E. coli* imp4213 cells. Thanks to the presence of biphenyl groups in the HM **LEGO-4** possesses fluorescent properties (Suppl. Fig. 7). Thus, mean fluorescence intensity of its emission maxima (λ_max_ ± 5 nm) in the supernatant and pelleted cells, respectively, was used as a measure of LEGO-LPPOs binding to the cells. For that purpose, we solubilized all fractions in 96% ethanol to unify the conditions. We assumed that the fluorescence measured in the pellet fraction roughly corresponds to the number of molecules bound to bacterial cells, while in the supernatant the fluorescence corresponds to the free fraction of unbound **LEGO-4**.

**Fig. 10:**
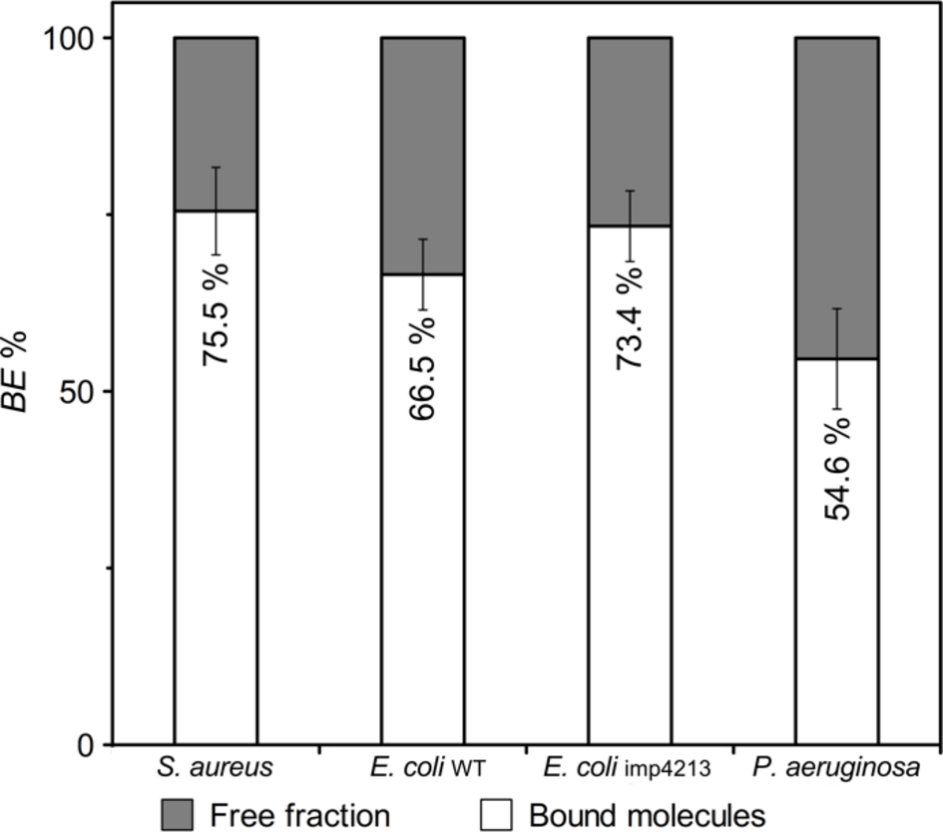
Binding efficiency of **LEGO-4** to *S. aureus*, *E. coli* CCM 3954 (WT), *E. coli* imp4213 cells, and *P. aeruginosa* as quantified by associated fluorescence intensity. We show an average (± SD) from two experiments performed in duplicates.

We measured the emission spectra of **LEGO-4** in all samples (Suppl. Fig. 7) after 15 min of incubation in the concentration of 8 mg/L. This timing corresponds to the state of highly permeabilized (more than 50%) *S. aureus* and *E. coli* imp4213 membranes (Fig. 7D,L), partially permeabilized (around 50%) *P. aeruginosa* membranes (Fig. 7P) and poorly permeabilized (less than 10%) *E. coli* CCM 3954 membranes (Fig. 7H). We found out that **LEGO-4** can bind to bacterial cells within 15 min after addition (Fig. 10). The molecules were least able to bind to *P. aeruginosa* with 54.6% efficiency and *E. coli* CCM 3945 with 66.5% efficiency, whereas it bound to higher extent to *S. aureus* CCM 4223 and *E. coli imp*4213 with comparable efficiency of 75.5% and 73.4%, respectively.

The results of this experiment show that different affinities for the entire bacterial species may also be crucial for the mechanism of action of the **LEGO-4** molecule and are consistent with the results of the experiments with PI and time-kill assay, which showed lower susceptibility of wild type *E. coli* and *P. aeruginosa.*

## Discussion

In this study, we selected four LEGO-LPPOs from a series of candidate antimicrobial compounds^18^ and carried out a structure–activity study, i.e. we compared how their antibacterial activity and action mechanism is influenced by their structure. Selected LEGO-LPPOs (Fig. 1, Table 1) specifically differ in the number of carbons in the central aliphatic linker (3, 4 or 6 carbon units) or in the structure of their hydrophobic modules bearing an alkyl chain (**LEGO-1** and **LEGO-2**), adamantylethyl (**LEGO-3**), or a biphenylethyl (**LEGO-4**), respectively.

Despites the structural differences, the overall activity and mode of action is very similar for all compounds. All LEGO-LPPOs exhibit bactericidal mode of action (MBC was either equal or twice the MIC, Suppl. Table 2). This is caused by irreversible membrane damage due to pore formation (Suppl. Fig. 8) and membrane potential decrease of bacterial cells^18^. The inoculum effect, also described as collective bacterial tolerance, is a significant issue in antibiotic treatment, because it can reduce its effectiveness^27^. It is a universal phenomenon described for most of the membrane active compound classes (antimicrobial peptides, peptidomimetics, toxins, etc.)^28,29^. Of note, no substantial MIC-advancement point was observed for tested LEGO-LPPOs. The sensitivity of bacteria to LEGO-LPPOs decreases uniformly, with a tenfold increase in bacterial numbers leading to less than twofold increase in MIC (Fig. 2, Suppl. Fig. 2), demonstrating a moderate inoculum effect and suggesting that the interaction between LEGO-LPPOs and bacterial cells is non-specific and is rather influenced by the ratio of molecules to bacterial cells. There may be a threshold number of LEGO-LPPOs that can disrupt the membrane enough to kill the bacteria. The steeper is the increase in MIC with inoculum size, the narrower the interval of the LEGO-LPPOs-to-cell ratio in which the molecules are most effective. The more specific the interaction between the antibiotic and bacterial cell is, the higher the inoculum effect could be^22^. Another cause of high inoculum effect could also be the production of degrading enzymes by the bacterial strain or the change of environment in the proximity of cells^30^, but this is not the case of studied LEGO-LPPOs. The IE is most strongly pronounced e.g. for β-lactamase producing bacteria exposed to β-lactams; however, it also has been observed for other classes of antimicrobials, including glycopeptides, macrolides, aminoglycosides, fluoroquinolones, daptomycin, and folate pathway inhibitors^25^. Using LEGO-LPPOs the IE was strongest in the case of *E. coli* imp4213 with an impaired outer membrane. Lack of significant differences in IE among different LEGO-LPPOs suggests that the action mechanism is similar for all four tested compounds.

Our data show that the antibacterial activity of LEGO-LPPO molecules is affected by the length of linker module—**LEGO-1** with the longest linker (six carbons, Fig. 1B) exerts higher antimicrobial activity than **LEGO-2–4** with three- to four-carbon linker modules. **LEGO-1** is highly structurally related to **LEGO-2**; it differs only in two carbon atoms shorter linker module (HM modules are the same). The additional two carbons decrease MIC values two- or four-times (all tested strain considered). Based on the membrane permeabilization kinetics (PI assay), similar MIC values against tested bacterial strains, and the results obtained on liposomes, where **LEGO-1** is also the most active, we can assume that the linker module length does not only affect selectivity but the general ability to disrupt membranes as it influences the overall hydrophobicity of the molecule (Table 1).

We also noticed that kinetics of PI influx into permeabilized cells varies more among the tested bacterial strains than among the LEGO-LPPOs used. In Gram-positive cells it was a rapid action while in Gram-negative cells there appeared two distinct phases of PI influx to the cells (supported by the values of Hill number, Suppl. Tables 3 and 4). In *S. aureus*, the increase in PI fluorescence is a very rapid, non-cooperative process which reaches its maximum within 10 min after any LEGO-LPPO addition. In *P. aeruginosa*, the increase in fluorescence intensity is slower and reaches a maximum after approximately 30 min, and in the wild-type *E. coli*, no maximum was reached at all during the 120 min of measurement. Of note, the intensity of permeabilization of *E. coli* imp4213 with increased outer membrane permeability (confirmed by higher NPN fluorescence intensity before LEGO-LPPO treatment, Suppl. Fig. 9), is comparable to *S. aureus*. The most pronounced biphasic character of permeabilization was also observed in this *E. coli* strain. Results of our other structure–activity study with related set of LEGO-LPPOs show the similar trend^31^. We hypothesize that the biphasic kinetics observed for some LEGO-LPPO concentrations on Gram-negative strains could be explained by initial accumulation and retention of LEGO-LPPOs molecules in the outer membrane, from where they are subsequently released and can form pores in the cytoplasmic membrane. Another possibility is that LEGO-LPPOs form two distinct pore phenotypes with varying numbers of subunits which appear in time. We see a correlation between the PI kinetics results and the outcomes of a time-kill assay, in which *E. coli* CCM 3954 experiences a slower killing compared to *E. coli* imp4213 (Fig. 9). After 24 h it resulted in the same outcome—no living cells were observed at all. In this assay we also noticed secondary decrease in the number of living cells of *E. coli* imp4213 which corresponded with the second phase of kinetics observed using PI (Fig. 9A, function *f*_*2*_). Although currently we are not able to distinguish whether the cell permeabilization is realized by a high number of small pores or few large oligomeric pores, the experiments with ATNS/DPX-loaded liposomes suggested that a combination of these two mechanisms is possible. Hill number around 1 signifies non-cooperative pore formation, which corresponds with increasing number of small pores. This was calculated for Gram-positive strains and *E. coli* imp4213 (Suppl. Table 4). In contrast, biphasic behavior of LEGO-LPPOs in Gram-negative strains and Hill number > 1 suggest cooperative behavior and therefore possible interaction of more LEGO-LPPOs molecules inserted into the membrane (Suppl. Table 3).

We were faced with the question which feature of the bacterial cell is the cause of this difference and whether the molecules, despite different MIC values, kill bacteria by the same action mechanism. In fact, in addition to higher MIC values (Table 2), significantly slower permeabilization kinetics (Fig. 7) can be noticed in Gram-negative bacteria, even at concentrations that are equivalent to or higher than the MIC*.* The difference in the effective concentration may not be that big, but the killing rate in Gram-positives is much faster. We hypothesized that the contrasting efficiencies are due to differences in phospholipid composition, which is known to affect the ability of antibiotic binding and form pores in the membrane, subsequently. The most sensitive strain studied was *S. aureus*, whose cytoplasmic membrane is composed of phosphatidylglycerol, lysylphosphatidylglycerol and cardiolipin^32^. In contrast, both *E. coli* and *P. aeruginosa* have phosphatidylethanolamine as the major component of their cytoplasmic membrane. Based on these results, one might predict an inhibitory effect of PE, which has been previously described in the literature for some membrane-active peptides^33^. Later, we show that this hypothesis was not right in our case.

Conductance measurements on planar lipid membranes confirmed pore-forming activity for molecules **LEGO-1–4** on all the types of tested phospholipid compositions—DPhPG, PE/PG (2:1) and PG/PE (2:1) (Suppl. Fig. 8). The pores exhibited a variety of phenotypes (dynamic, stably open, gradual membrane rupture—for typical current recordings see Suppl. Fig. 8A) with wide conductance range from 23 pS to 41,000 pS (PE/PG, 2:1) and from 12 to 10,000 pS (PG/PE, 2:1) (Suppl. Fig. 8B,C). Thus, the range of pore conductances was broader in case of more electroneutral membranes (PE/PG, 2:1). The exact stoichiometry of the pores is not yet clear. It could resemble varying number of monomers in oligomeric pores, which was described for LPPO II^15^. These results are consistent with other, structurally related members of the studied generation of LEGO-LPPOs^31^. Thus, we hypothesize that both small pores and larger multimeric pores can be made by LEGO-LPPOs in artificial as well as cytoplasmic membrane. Small monomolecular membrane ruptures cause immediate leakage upon contact with the cytoplasmic membrane in Gram-positive bacteria. In Gram-negatives, the penetration of LEGO-LPPO molecules is affected by the presence of outer membrane which is demonstrated by an initial slower phase of permeabilization followed by a second phase when gradually formed larger oligomeric pores are present in the membrane. We have confirmed in experiments with fluorescent probe NPN that **LEGO-LPPO** can disrupt outer membrane of *E. coli* CCM 3954, **LEGO-1** being the most effective (Suppl. Fig. 9). In this case, the initial disruption by small pores might only take place at the outer membrane. In contrast, the highly permeable outer membrane of *E. coli* imp4213 cannot be more permeabilized (Suppl. Fig. 9) and lets the LEGO-LPPO molecule trough directly to the cytoplasmic membrane. At some concentrations, membrane damage may be observed in both ways (small pores of initial phase and bigger pores gradually formed after several minutes) due to the more permeable outer membrane of *E. coli* imp4213.

The cause of the reduced susceptibility and slower action on Gram-negative strains may also be simply the physical presence of an outer membrane as the obstacle that hinders the passage of molecules to the cytoplasmic membrane. We have previously shown that lipopolysaccharide (LPS) has an inhibitory effect on liposome lysis induced by LPPO II^34^. Given their amphipathic nature, the passage of LEGO-LPPOs across outer membrane is likely to be lipid-mediated, as it is the case with antimicrobial peptides^35^. Other explanation could be some specific interactions of LEGO-LPPOs with one of the outer membrane components—e.g. LPS. This would reduce number of LEGO-LPPO molecules interacting with the target site—cytoplasmic membrane, and thus hindering their activity. We calculated the binding efficiency of fluorescent molecule **LEGO-4** to all four bacterial strains tested (Fig. 10). We obtained comparable results for binding of **LEGO-4** to *S. aureus* and *E. coli* imp4213 cells, binding ability to *E. coli* and *P. aeruginosa* cells was approximately 10–20% weaker. The key role of the intact outer membrane in sensitivity to LEGO-LPPOs is also clearly demonstrated by MIC—the values for the mutant *E. coli* strain are similar to values for Gram-positive bacteria. Therefore, it seems that the different affinity of LEGO-LPPO for Gram-positive and Gram-negative bacteria directly affects the sensitivity of strains in terms of MIC. Whether the outer membrane acts only as a physical barrier or whether LEGO-LPPOs interact specifically with it needs to be investigated further.

Given that the membrane phospholipids represent the target site of LEGO-LPPOs, we used liposomes with two distinct lipid compositions to assess its effect on membrane permeabilization induced by **LEGO-1–4**. The two mixtures roughly resembled membrane composition and/or charge of Gram-positive (PG/PE, 2:1) or Gram-negative (PE/PG, 2:1) bacterial strains. The DPX/ANTS permeability ratio (Fig. 3), which is indicative of ion selectivity of the membrane pores and mechanism of liposome leakage, is described by the parameter α. The α value for all studied LEGO-LPPO molecules ranged from 0.3 to 0.8. This corresponds to an “ANTS selective” gradual release of liposomal content which can be associated with the formation of smaller membrane pores. However, it has been postulated that the α value in this range does not clearly determine the exact mechanism and does not allow further specification of this behavior^36^. The results can be interpreted as a combined process in which a certain fraction of liposomes undergoes “all-or-none” lysis and another fraction undergoes slow, gradual rupture. Our results, which admit the possibility of anion-selective pores, could suggest the formation of large pores. Given the estimated diameter of the ANTS and DPX molecules (both approx. 0.4 nm) that passed through the pores in the liposome membranes, the size of LEGO-LPPOs pores can be estimated to be at least 0.4 nm^37^. Using ANTS-DPX-loaded liposomes, we also observed different levels of lysis induced by the action of individual LEGO-LPPOs. The highest fluorescence intensity (*I*) values were measured in the case of the **LEGO-4** molecule, the lowest values were observed for the **LEGO-2** (Fig. 4).

Of note, liposomes with higher content of PE (resembling Gram-negatives) showed higher permeabilization rate by LEGO-LPPOs. This was also observed and confirmed on liposomes filled with carboxyfluorescein^31^. Using the requenching method, we determined that the phospholipid composition did not change the action mechanism. However, in PE/PG liposomes, the lysis rate was higher in the case of all tested compounds. These findings are consistent with the conductivity measurements on planar membranes and were also confirmed in a in silico analysis, which predicts significantly higher effect of **LEGO-1** on the membrane systems with DOPE as the major phospholipid. The results showed preferential contact of **LEGO-1** bearing +6 charge with the anionic phospholipid DOPG. The amino group of DOPE is bound selectively by O1/O3 atoms of **LEGO-1**. It signifies that such interacting amino group of DOPE is forced to be positioned deep in the membrane, on the level of phosphonate group of **LEGO-1**. This is actually one or two carbon atoms deeper than the phosphate groups of DOPE or DOPG (cf. Fig. 5). We propose that the overall lower number of contacts among **LEGO-1** molecules and zwitterionic phospholipid DOPE stems from an electrostatic repulsion. This could be destabilizing for the membrane and might explain the increased permeabilization of PE/PG membranes induced by **LEGO-1** in vitro. The same principle of electrostatic repulsion may apply in the interaction of **LEGO-1** with positively charged phospholipid lysylphosphatidylglycerol in the membrane of *S. aureus*, which is highly sensitive to **LEGO-1** action. We are planning to further test this interaction in our future research on in vitro systems. It is expected that molecules such LEGO-LPPOs tend to make larger complexes in the membranes that are more ordered and less hydrated which is the case of PE/PG compared to PG/PE membrane^31^. Such an environment supports the lateral separation of membrane inserted molecules and therefore increases the chance of inter-LEGO-LPPOs interaction leading to formation of larger DOPE-dominated complexes.

## Methods

### Synthesis of LEGO-LPPOs

Synthesis of **LEGO-1**, **-2** and **-3** was already published^18^. **LEGO-4** was synthesized according to scheme 1 presented in Supplementary material.

### Bacterial strains

To compare LEGO-LPPOs action mechanism, we selected standard reference bacterial strains *Staphylococcus aureus* CCM 4223, *Pseudomonas aeruginosa* CCM 3955 and *Escherichia coli* CCM 3954 (Czech Collection of Microorganisms, Faculty of Science, Masaryk University) as representatives of both Gram-positive and Gram-negative bacteria. Representatives of these genera are also clinically relevant nosocomial or opportunistic pathogens. To better understand and describe the role of intact outer membrane, we also included *Escherichia coli* imp4213 in all experiments^38^. Chromosome of *E. coli* imp4213 contains mutations in lptD gene coding for a protein involved in LPS transport-assembly system, which increases the permeability of outer membrane^21^. All tested strains were stored in 15% glycerol solution at − 70 °C.

### Cell cultivation

Bacteria from cryotubes were inoculated on Luria Bertani agar plates, cultivated overnight at 37 °C and stored at 4 °C for no longer than 4 weeks. Cells were cultivated aerobically (37 °C, 180 RPM) from overnight liquid culture in fresh Mueller Hinton (MH) or Luria Bertani (LB) broth until the suspension reached cell density of approximately 10^8^ CFU/mL. We spectroscopically monitored bacterial growth by measuring optical density at 450 nm. The culture was then harvested by centrifugation (8000*g*, 25 °C, 15 min), directly diluted or resuspended in relevant buffer or fresh medium to the appropriate cell density which varied according to the individual protocols described below. Antibiotic susceptibility testing is standardly set as minimum inhibitory concentration (MIC) for bacterial suspension of inoculum density 5 × 10^5^ CFU/mL, while some of the experiments (PI assay, time-kill assay, and determination of binding efficiency) were performed with suspensions of higher densities (4 × 10^7^ CFU/mL) in order to get enough signal. Taking account also the inoculum effect (see below), we adjusted the LEGO-LPPOs concentration in each protocol accordingly so the antimicrobial activity would not be lowered. These results need to be considered when interpreting the data.

### Determination of MIC and MBC values and the effect of inoculum size

Antibacterial activity of all tested molecules against selected bacterial strains was evaluated according to the standard broth microdilution protocol^39^. Determination of MIC was performed in plastic 96-well microtitration plates. Cell cultivation was performed in MH broth (37 °C, 180 RPM), until cell density of 5 × 10^8^ CFU/mL was reached. The final concentration of cell suspension in each well was 5 × 10^5^ CFU/mL. Concentration range of tested LEGO-LPPOs was between 128 and 0.06 mg/L. LEGO-LPPOs were thawed and diluted from a stock solution of 20 mg/mL (in ddH_2_O) to MH broth approximately two hours before the experiment. The total volume of each well of the microtitration plate was finally 100 µL and contained MH broth, cell suspension and LEGO-LPPOs in appropriate concentration. The MIC values were then determined after 24 h of incubation at 37 °C as the lowest concentration that visibly inhibited growth.

We also assessed bactericidal activity of LEGO-LPPOs (expressed as minimum bactericidal concentration, MBC) by plating 5 μL drops of bacterial suspension from each microplate well on MH agar plate and incubating for another 24 h in 37 °C. MBC was determined as the concentration that prevented growth irreversibly, i.e. there were no bacterial colonies visible on the agar plate. The presented results are average values of two experiments performed in triplicates.

To test the effect of size of the inoculum on the value of MIC, aliquots of bacterial suspension of cell density ranging from 10^8^ to 10^2^ CFU/mL were added to 96-well microtitration plate with serial two-fold dilutions of LEGO-LPPOs (*c* = 0.125–32 mg/mL) and incubated at 37 °C. After overnight incubation, for each inoculum size the MIC and MBC were determined as described above.

### Evaluation of the bactericidal effect of LEGO-LPPOs in time (Time-Kill Assay)

Bacterial suspension (*E. coli* CCM 3954 and *E. coli* imp4213) of 10^8^ CFU/mL cell density was centrifuged (8000*g*, 25 °C, 15 min), washed in a buffer (10 mM HEPES, 0.5% glucose, pH 7.2, filtrated) and then resuspended to the final cell density of approximately 10^7^ CFU/mL. The time-kill assay was performed in sterile tubes with the cell suspension, where **LEGO-4** was added to final concentration of 8 mg/L. The suspension was then incubated (37 °C, with shaking) with the compound. At indicated time intervals (t = 5, 10, 30, 60, 120 min and overnight), CFU was determined. The final CFU/mL was calculated as average from triplicates of at least two dilutions.

### Determination of hemolytic activity

The hemolytic activity of LEGO-LPPOs was determined by measuring the release of hemoglobin from human red blood cells. The study was approved by the Ethics Commission of the Charles University. All procedures were performed in accordance with the relevant guidelines and regulations and the volunteer had signed the informed consent. Blood was aseptically taken from a volunteer donor to EDTA collection tubes and was centrifuged at 1000*g*, 4 °C, and 10 min. The supernatant was discarded, and erythrocytes were washed with 150 mM NaCl and then resuspended to a concentration of 2% (v/v) in 150 mM NaCl. This erythrocyte suspension was incubated with serial dilutions of LEGO-LPPOs (0.5–200 mg/L) dissolved in 150 mM NaCl at 37 °C for three hours. After incubation, the mixture was centrifuged (1000*g*, 4 °C, 5 min), and the absorbance of the supernatant was measured at 540 nm. Erythrocytes incubated with Triton X-100 (1%, v/v) in 150 mM NaCl served as the positive control (100% hemolytic activity), while erythrocytes incubated with 150 mM NaCl only served as the negative control (0% hemolytic activity). Hemolytic activity of LEGO-LPPOs was expressed as the concentration causing 50% lysis of erythrocytes (HC_50_).

### Liposome preparation and leakage assay

To clarify the LEGO-LPPOs lysis mechanism, we used lipid vesicles of diameter 100 and 1000 nm as an artificial membrane model system. Measurements on liposomes of different sizes can be used to distinguish more precisely between mechanism and selectivity. When measuring LUV_100_ lysis, it is easier to distinguish “all-or-none” from gradual leakage, as smaller liposomes are more quickly completely emptied, whereas LUV_1000_ allows for a more accurate determination of the α parameter and pore selectivity, especially if α > 1^40^. In the case of LEGO-1–4, LUV_100_ give the same range of α values as LUV_1000_.

Liposomes were prepared from synthetic phospholipids (Avanti Polar Lipids) according to the previously described protocol^41^. Namely, lipids 1,2-dioleoyl-sn-glycero-3-phospho-(1′-rac-glycerol)—DOPG and 1,2-dioleoyl-sn-glycero-3-phosphoethanolamine—DOPE, in a ratio 2:1 (m/m) and 1:2 (m/m) (Avanti Polar Lipids, inc., USA) were used to mimic cytoplasmic membrane of Gram-positive and Gram-negative bacteria, respectively.

Phospholipids were diluted in chloroform and mixed in a glass tube to final concentration 10 mg/mL and then evaporated under the nitrogen flow to form a thin film on the walls. After that, inner buffer (50 mM TRIS, 150 mM NaCl, 15 mM ANTS 30 mM DPX, pH 7.4) was added and the tube was shaken, until all phospholipids were washed from the walls and multilamellar vesicles were formed. The extruder (Avanti Polar Lipids) was then used to form liposomes of defined size by repeated extrusion of multilamellar vesicles through 1000 or 100 nm polycarbonate filters (Whatman).

The free fluorescent probe was removed by gel filtration in a column filled with a Sephadex G-50 and eluted with 50 mM TRIS and 150 mM NaCl (pH 7.4) outer buffer. With this buffer, final phospholipid concentration of 10 µM was obtained (according to the measured content of inorganic phosphate).

Requenching assay was performed as described previously^40,42^. LEGO-LPPOs from 1 mg/mL stock solution were added into 1.5 mL of liposome suspension to the final concentrations ranging from 0.25 to 64 mg/L. This was followed by incubation of the mixture in the dark for 90 min at room temperature.

Fluorescence of the incubated samples was measured using FluoroMax-3 spectrofluorometer (Jobin Yvon, Horiba), in 10 × 10 mm quartz cuvettes. Then DPX was added to each cuvette to reach the final concentration of 93 mM. Total quenching was determined. At the end of the measurement, Triton X-100 in the concentration of 1% was added to reach maximum level of fluorescence. With the measured values, we were able to calculate the level of quenching inside (*Q*_*in*_) and the fraction of ANTS outside (*f*_*out*_) and therefore determine the mechanism of leakage according to the requenching method^42^.

The method allows us to describe the mechanism of leakage as either a graded process of content release or an all-or-none mechanism. The leakage can be described as follows (Eq. 1):

1 $$Q_{in} = \left\{ {\left[ {1 + K_{D} \left[ {DPX} \right]_{0} \left( {1 - f_{out}^{ANTS} } \right)^{\alpha } } \right]\left[ {1 + K_{S} \left[ {DPX} \right]_{0} \left( {1 - f_{out}^{ANTS} } \right)^{\alpha } } \right]} \right\}^{ - 1}$$

If *Q*_*in*_ observed for different LEGO-LPPO concentrations is independent of *f*_*out*_, then the mechanism would be considered as all-or-none. If *Q*_*in*_ increases with *f*_*out*_ then the leakage is considered to be graded. In the Eq. (1) [DPX]_*0*_ is the initial concentration of DPX in the vesicles and α is the parameter, that describes selectivity. It is defined as the ratio of the rates of release of DPX and ANTS.

We show representative data sets from two experiments performed in duplicate.

Using Fityk software^43^, the dose–response curves of liposome lysis were fitted to the function:

2 $$\Theta = \frac{{\left[ C \right]^{n} }}{{(K_{A} )^{n} + \left[ C \right]^{n} }}$$

where *Θ* is the relative amount of ANTS that leaked the liposomes (*f*_*out*_), [*C*] is LEGO-LPPO concentration, *K*_*A*_ is the LEGO-LPPO concentration producing the half-maximal increase of *f*_*out*_, and *n* is the Hill coefficient.

### Cell permeabilization assay

The cell suspension of approximately 10^8^ CFU/mL was centrifuged (8000*g*, 10 min, 20 °C), and then washed in a buffer (10 mM HEPES, 0.5% glucose, pH 7.2, filtrated) and resuspended to the final cell density of 10^7^ CFU/mL. Propidium iodide (PI) from 20 mM stock (in DMSO) was added to the cell suspension to the final concentration of 10 µM and mixed properly. The suspension was then pipetted to the plastic 96-well microtitration plate with serial two-fold dilutions of LEGO-LPPOs (*c* = 0.125–32 mg/mL) and mixed. We used MicroMax-3 (Jobin Yvon, Horiba) operated by FluoroMax-3 spectrofluorometer (Jobin Yvon, Horiba) to detect the fluorescence intensity (the increase of which is considered as PI uptake) in each well of the microtitration plate. PI fluorescence measurement was started immediately and continued for at least 150 min for each bacterial strain-compound combination. The measurement was done at following conditions: λ_ex_ = 515 nm, λ_em_ = 620 nm. We used optical filters for suppression of scattered light (Omega Optical filters 3RD500-530 and 3RD570LP in excitation and emission paths, respectively). Both excitation and emission slits were set to 15 nm.

Melittin (1 µM from 1 mM stock in ddH_2_O) was used as a positive control of membrane permeabilization and set as the maximum value of fluorescence (*I*_*max*_) for each experiment repetition. Non-treated cell suspension was used as fluorescence baseline (*I*_*cells*_). For each well, intensity of PI fluorescence (%) was calculated (Eq. 3):

3 $$PI\; \left( \% \right) = \frac{{\left( {I_{LEGO - LPPO} - I_{cells} } \right)}}{{\left( {I_{max} - I_{cells} } \right)}} \times 100$$

For each bacterial strain-compound combination the protocol was performed at least two times. We show average values of normalized fluorescence intensity calculated from triplicates. Where appropriate, before reaching the plateau phase, the rate of lysis was determined by fitting the curves to the linear function *y* = *v* × *t*, where *t* is the time and *v* is the rate of lysis expressed as the percentage of maximum lysis per minute.

Using Fityk software^43^, the time courses of propidium iodide entry to the cells were fitted to the function:

4 $$f\left( t \right) = \alpha \left( {1 - \exp \left( { - \frac{t}{\tau }} \right)} \right)^{n}$$

where *t* is the time, α is the amplitude of the effect, *τ* is the time constant of the effect, and *n* is the coefficient that expresses positive (*n* > 1) or negative (*n* < 1) cooperativity of the leakage, respectively^44^. The functions with *n* < 1 are further designated as “non-cooperative” and functions with *n* > 1 as “cooperative”. A single function or combination of several functions can be used to describe the kinetics, suggesting a multi-modal action.

### Determination of LEGO-LPPOs affinity to bacterial cells

We used fluorescent properties of biphenyl hydrophobic modules of **LEGO-4** to quantify its fluorescence in the samples of cell suspensions and therefore to determine its binding efficiency (*BE*, Eq. 5) to bacterial cells of *S. aureus* CCM 4223, *E. coli* CCM 3954 and *E. coli* imp4213. The freshly grown cell culture with density of approximately 10^8^ CFU/mL was centrifuged (8000*g*, 10 min, 20 °C), washed with a buffer (10 mM HEPES, 0.5% glucose, pH 7.2, filtrated), resuspended to the final cell density of approximately 10^7^ CFU/mL, and divided into 10 mL aliquots. LEGO-4 (1 mg/mL in ddH2O) was then added to the final concentration of 8 mg/L and the sample was incubated at 37 °C for 15 min. After centrifugation (8000*g*, 30 min, 10 °C), the samples (200 μL aliquots) were transferred to 1.4 mL 96% ethanol to avoid different fluorescence of **LEGO-4** in individual environments. The final ethanol concentration was 84%. The fluorescence intensity of supernatant (*I*_*SNA*_, Eq. 5), pellet (*I*_*PA*_) and original labelled cell suspension (*I*_*CA*_) was determined in 10 × 10 mm quartz cuvette. We used FluoroMax-3 spectrofluorometer (Jobin Yvon, Horiba) to record the emission spectra of **LEGO-4** after excitation of each sample at 265 nm (Suppl. Fig. 7). The average of emission intensities between 315 and 325 nm was considered in the calculations. The binding efficiency (*BE*) was calculated according to Eq. (5). As a negative control, untreated cell suspension (intensity *I*_*CB*_) was processed following the same protocol resulting in untreated supernatant (*I*_*SNB*_) and untreated pellet (*I*_*PB*_). Fluorescence of untreated samples (*I*_*SNB*_, *I*_*PB*_, *I*_*CB*_) was subtracted as a background from each treated fraction. Measured intensities were corrected for the dilution state (*D*) of each fraction corresponding to the ratio of the total volume of supernatant (*V*_*SN*_) and the total volume of pellet (*V*_*P*_, Eq. 6).

5 $$BE \left( \% \right) = \frac{{\left( {I_{P} } \right)}}{{\left( {I_{P} + I_{SN} } \right)}} \times 100,\quad {\text{where}}\;\;I_{P} = \frac{{I_{PA} }}{D} - I_{PB} \;\;{\text{and}}\;\;I_{SN} = I_{SNA} - I_{SNB}$$

6 $$D = \frac{{V_{SN} }}{{V_{P} }}$$

As a positive control of highly efficient binding of a fluorophore to membranes, we used 1,6-diphenyl-1,3,5-hexatriene (DPH). In this case, the cells were labelled in 10 µM solution of DPH for 30 min at 37 °C in the dark. The following protocol was the same as described above. Excitation wavelength was 350 nm and average emission intensities between 455 and 465 nm were used in calculations. We were able to detect 90 ± 3% of total DPH fluorescence (almost 100% was expected) in the pellet fraction. The proportion of fluorescence detected in pellet was considered as a control for centrifugation reliability.

### *In silico* simulation of LEGO-LPPOs interaction with cytoplasmic membrane

Fully-atomistic MD simulations were performed using the GROMACS 2023.3 software suite^45^. Initially, lipid bilayers composed of DOPG and DOPE phospholipids (128 lipids, 64 in each leaflet), along with 150 mM potassium and chloride ions, were generated and hydrated with ~ 6,300 water molecules (see Supplementary Table 3 for detailed compositions). Additional ions were added to neutralize each system. The membranes were prepared using the CHARMM-GUI web server and equilibrated for 500 ns at 310 K following the standard CHARMM-GUI protocol (see Suppl. Table 5)^46^. After equilibration, sixteen **LEGO-1** molecules, along with neutralizing chloride anions, were symmetrically added to the water phase adjacent to each membrane, resulting in a 1:8 **LEGO-1**-to-lipid ratio. The systems were then simulated for an additional 2000 ns, during which the **LEGO-1** molecules interacted with and incorporated into the lipid bilayers. Equilibration was achieved after 500 ns, as determined by monitoring **LEGO-1**-lipid contacts. We utilized the fully-atomistic CHARMM36 force field^47^. Force field parameters for **LEGO-1** molecule were obtained using the CHARMM-GUI web server^48^. We assumed a full protonation state of **LEGO-1** with the total charge of +6. TIP3 parameterization was used for water^49^. Trajectory analysis was conducted using standard GROMACS tools and custom Python scripts. VMD was used for trajectories visualization^50^ and PyMOL was used for snapshots generation.

## Supplementary Information

Supplementary Material 1.

## Data Availability

The datasets used and/or analyzed during the current study are available from the corresponding author on request.
