# Supplementary material for: LEGO-Lipophosphonoxin membrane activity is enhanced by presence of phosphatidylethanolamine but hindered by outer membrane
Source: Sci Rep. 2025 Jan 7;15:1206. doi: 10.1038/s41598-024-83205-w (PMC11707287; doi:10.1038/s41598-024-83205-w)
Supplement: Supplementary file 1 — Supplementary Material 1. [file 41598_2024_83205_MOESM1_ESM.docx]

**Supplementary material**

LEGO-Lipophosphonoxin membrane activity is enhanced by presence of phosphatidylethanolamine but hindered by outer membrane

Hana Brzobohatá^1^, Milica Dugić^1^, Viktor Mojr^2^, Nitjawan Sahatsapan^2^, Ivana Kóšiová^2^, Tomáš Křížek^3^, Tereza Dolejšová^1^, Petra Lišková^1^, Lukasz Cwiklik^4^, Dominik Rejman^2*^, Radovan Fišer^1*^, Gabriela Mikušová^1*^

^1^Department of Genetics and Microbiology, Faculty of Science, Charles University, Viničná 5, 128 00, Prague, Czech Republic

^2^Institute of Organic Chemistry and Biochemistry, Czech Academy of Sciences v.v.i., Flemingovo náměstí 2, 166 10 Prague 6, Czech Republic

^3^Department of Analytical Chemistry, Faculty of Science, Charles University, Charles University, Hlavova 8, 128 00, Prague, Czech Republic

^4^J. Heyrovský Institute of Physical Chemistry, Czech Academy of Sciences v.v.i., Dolejškova 3, 182 23 Prague, Czech Republic

*Corresponding authors: Dominik Rejman ([rejman@uochb.cas.cz](mailto:rejman@uochb.cas.cz)), Radovan Fišer (fiserr@natur.cuni.cz), Gabriela Mikušová (seydlova@natur.cuni.cz)

**Synthesis of LEGO-LPPOs**

Synthesis of **LEGO-1**, **-2** and **-3** was already published^1^. **LEGO-4** was synthesized according to scheme 1.

**Scheme 1**. Synthesis of **LEGO-4**.

**General conditions**

Unless stated otherwise, all used solvents were anhydrous. TLC was performed on silica gel pre-coated aluminium plates TLC Silica gel 60 F₂₅₄ (Supelco), and compounds were detected by UV light (254 nm), by spraying with 1% solution of ninhydrine to visualize amines, and by spraying with 1% solution of 4-(4-nitrobenzyl)pyridine in ethanol followed by heating and treating with gaseous ammonia (blue color) to visualize phosphonates. Preparative column chromatography was carried out on silica gel (40–63µm, VWR Chemicals), and elution was performed at the flow rate of 60–80 mL/min. The concentrations of solvent systems are stated in volume percents (%, *v*/*v*). Mass spectra were recorded on LTQ Orbitrap XL (Thermo Fisher Scientific) using ESI ionization. NMR spectra were measured on Bruker AVANCE III™ HD 400 MHz (^1^H at 400.1 MHz, ^13^C at 100.6 MHz), Bruker Avance III™ HD 400 MHz Prodigy (^1^H at 401.0 MHz, ^13^C at 100.8 MHz), Bruker Avance III™ HD 500 MHz (^1^H at 500.0 MHz, ^13^C at 125.7 MHz) and JEOL JNM-ECZR 500 MHz (^1^H at 500.2 MHz, ^13^C at 125.8 MHz) spectrometers. Chemical shifts (in ppm, δ scale) were referenced to TMS as internal standard, coupling constants (*J*) are given in Hz.

**Mono 2-(biphenyl-4-yl)ethyl vinylphosphonate 2**

A mixture of mono methyl vinylphosphonate (2.50 g; 20.5 mmol), 2-(biphenyl-4-yl)ethanol (5.99 g; 30.2 mmol) a 4-methoxy-1-*N*-oxidopyridine (MOP) (512 mg; 4.09 mmol) was co-evaporated with toluene (100 ml) and pyridine (100 ml) and dissolved in pyridine (160 ml). 2-Chloro-5,5-dimethyl-1,3,2-dioxaphosphinane 2-oxide (NEP) (9.44 g; 51.1 mmol) was added and the reaction mixture was stirred under argon atmosphere at rt for 3 h. The reaction mixture was concentrated in vacuo, dissolved in toluene (400 mL) and filtered over cellite. The filtrate was washed with 2 mol.l^-1^ TEAB (400 ml). Aqueous phase was washed with toluene (400 ml). Combined organic phases were dried over sodium sulfate and concentrated in vacuo. Intermediate mixed diester was obtained by chromatography on silica gell using linear gradient of acetone in toluene was dissolved in diethyl ether (200 mL), filtered over cellite, concentrated in vacuo, dissolved in 60% aqueous pyridine (180 mL) and stirred at 60 °C overnight. The reaction mixture was concentrated in vacuo, dissolved in ethanol (200 mL) and passed through a column of DOWEX 50 H^+^ (200 ml). The column was washed with ethanol (200 ml). The titled product was obtained after evaporation of ethanol in 95% overall yield (5.61 g; 19.5 mmol) in the form of white amorphous solid. ^1^H NMR (400 MHz, DMSO-*d*_6_) δ 7.68–7.59 (m, 2H), 7.55 (d, *J* = 7.3 Hz, 2H), 7.44 (t, *J* = 7.6 Hz, 2H), 7.37–7.25 (m, 3H), 6.11–5.38 (m, 3H), 3.79 (brs, 2H), 2.81 (brs, 2H). ^13^C NMR (101 MHz, DMSO-*d*_6_) δ 140.15; 138.54; 137.86; 129.46; 128.88; 127.15; 126.50; 126.44; 63.77; 36.78. ^31^P NMR (162 MHz, DMSO-*d*_6_) δ 10.29. HRMS (ESI^+^): for C_16_H_18_O_3_P *m*/*z* calcd: 289.09881, found 289.09901.

**Butan-1,4-diyl bis(2-(biphenyl-4-yl)ethyl) bis(vinylphosphonate) 3**

A mixture of mono 2-(biphenyl-4-yl)ethyl vinylphosphonate **2** (7.86 g; 27.3 mmol), butan-1,4-diol (99%; 813 µl; 9,09 mmol) and MOP (455 mg; 3.63 mmol) was co-evaporated with pyridine (2×125 mL) and dissolved in the same solvent (90 mL). NEP (6.71 g; 36.4 mmol) was added and the reaction mixture was stirred under argon atmosphere at rt for 2 h. The reaction mixture was concentrated in vacuo, dissolved in chloroform (100 mL) and washed with 2 M TEAB (100 mL). Organic phase was dried over sodium sulfate and concentrated in vacuo. Titled product was obtained by chromatography on silica gel using linear gradient of acetone in toluene in 92% yield (5.28 g; 8.37 mmol,) in the form of white amorphous solid. ^1^H NMR (401 MHz, Methanol-*d*_4_) δ 7.65–7.57 (m, 8H), 7.46–7.40 (m, 4H), 7.40–7.36 (m, 4H), 7.36–7.31 (m, 2H), 4.44–4.31 (m, 4H), 4.10–3.91 (m, 4H), 3.40–3.31 (m, 12H), 3.11–2.97 (m, 12H), 2.53 (ddd, *J* = 19.8; 9.6; 4.9 Hz, 4H), 2.25–2.08 (m, 8H), 1.76–1.65 (m, 4H). ^13^C NMR (101 MHz, Methanol-*d*_4_) δ 141.86; 140.85; 138.01; 130.89–130.76 (m), 130.17–129.93 (m), 128.61–128.31 (m), 128.17–128.12 (m), 127.90–127.79 (m), 68.59 (d, *J* = 6.3 Hz), 67.53 (d, *J* = 6.6 Hz), 67.49 (d, *J* = 6.7 Hz), 50.96; 37.82; 37.31 (d, *J* = 6.4 Hz), 27.72 (d, *J* = 6.0 Hz), 27.68 (d, *J* = 6.1 Hz), 23.18; 21.38 (d, *J* = 140.1 Hz), 21.34 (d, *J* = 140.3 Hz). ^31^P NMR (162 MHz, Methanol-*d*_4_) δ 20.69. HRMS (ESI^+^): for C_34_H_37_O_6_P_2_ *m*/*z* calcd: 603.20599, found 603.20560.

**Butan-1,4-diyl bis(2-(biphenyl-4-yl)ethyl) bis((2-(bis(3-aminopropyl)amino)ethyl)-phosphonate) hexahydrochlorid 4 (LEGO-4)**

Michael addition of bis(3*-tert-*butyloxycarbonylaminopropyl)amine (0,93 g; 2,81 mmol) to **4** (590 mg; 936 µmol) and subsequent removal of Boc protecting groups was carried out according to described procedure^1^. Titled product was obtained in 63% overall yield (656 mg; 590 µmol) in the form of white amorphous solid. ^1^H NMR (401 MHz, Methanol-*d*_4_) δ 7.66–7.58 (m, 8H), 7.47–7.41 (m, 4H), 7.41–7.36 (m, 4H), 7.36–7.31 (m, 2H), 4.45–4.32 (m, 4H), 4.11–3.92 (m, 4H), 3.41–3.32 (m, 12H), 3.12–2.98 (m, 12H), 2.54 (ddd, *J* = 19.8; 9.6; 4.9 Hz, 4H), 2.26–2.09 (m, 8H), 1.77–1.66 (m, 4H). ^13^C NMR (101 MHz, Methanol-*d*_4_) δ 140.47; 139.45; 136.62; 129.50–129.36 (m), 128.77–128.53 (m), 127.22–126.91 (m), 126.77–126.72 (m), 126.51–126.40 (m), 67.19 (d, *J* = 6.3 Hz), 66.14 (d, *J* = 6.6 Hz), 66.10 (d, *J* = 6.7 Hz), 49.56; 36.42; 35.91 (d, *J* = 6.4 Hz), 26.32 (d, *J* = 6.0 Hz), 26.29 (d, *J* = 6.1 Hz), 21.79; 19.99 (d, *J* = 140.1 Hz), 19.95 (d, *J* = 140.3 Hz). ^31^P NMR (162 MHz, Methanol-*d*_4_) δ 28.57; 28.52. HRMS (ESI^+^): for C_48_H_75_O_6_N_6_P_2_ *m*/*z* calcd: 893.52178, found 893.52057; for C_48_H_76_O_6_N_6_P_2_ *m*/2*z* calcd: 447.26453, found: 447.26434.

**Handling of LEGO-LPPOs stock solutions**

LEGO-LPPOs (lyophilized powder) were diluted in ddH_2_O to the final concentration of 10 mg/mL or 20 mg/mL in the case of MIC determination. These stock solutions were then stored in a freezer for a maximum period of one month. Solution of lower concentrations were prepared by diluting LEGO-LPPOs stock (vortexed for a minute) in ddH_2_O just before performing the experiment.

**Cytotoxicity of LEGO-LPPOs**

We have previously determined and published (Do Pham et al., 2022) the cytotoxicity of **LEGO-1-3** tested on human non-tumorigenic hepatocytes (HepG2) using alamarBlue assay (Invitrogen) and expressed the values as CC_50_ (cytotoxic dose at 50% viability, mg/L). The cytotoxic concentrations of **LEGO-1-3** (Suppl. Table 1) were significantly higher than their MIC values (Do Pham et al., 2022)^1^.

Next, we performed MTT (4,5-[di](https://en.wikipedia.org/wiki/Numeral_prefix)methyl[thiazol](https://en.wikipedia.org/wiki/Thiazole)-2-yl)-2,5-diphenyltetrazolium bromide) cytotoxicity test on Balb/c 3T3 mouse fibroblasts cell line to evaluate LEGO-LPPOs effect on mitochondrial activity expressed as half-maximal inhibitory concentration (IC_50_, mg/L). The cryopreserved cells were taken out from the deep-freezer, left for 1 min at room temperature and then sterile transferred to a 25 cm^2^ flask with 10 ml of culture medium (DMEM, penicillin 100 U.mL^-1^, L- glutamine 2 mmol.L^-1^, streptomycin 100 mg.L^-1^, fetal calf serum 5%, neonatal calf serum 5%). The cells were stored in an incubator saturated with water vapour at 37 °C and in an atmosphere of 5% CO_2_, the medium was changed every 48 - 72 h. After reaching the monolayer, the cells were washed with sterile PBS (5 mL), released by incubation with 0.25% trypsin solution with EDTA (0.5 mL; 2-3 min, 37 °C), 5 mL of culture medium was added, and the samples were centrifuged (10 min, 300 g, room temperature). The pellet was resuspended in 20 mL of culture medium, and the cells were transferred to a 75 cm^2^ culture flask and further cultured. After reaching the monolayer, the cells were washed with sterile PBS (10 mL), released by incubation with 0.25% trypsin solution with EDTA (1 mL; 2-3 min; 37 °C), and resuspended in 10 mL of culture medium. The suspension was centrifuged (10 min; 300 g; room temperature). The pellet was resuspended in 10 mL of culture medium and the cells were used for the experiment.

Stock solutions of **LEGO-1**, **-2** and **-3** were prepared in DMSO so that the final concentration of DMSO in the medium was 0.5 % (v/v). Control cells were prepared by incubation with an appropriate volume of DMSO. Cell concentrations were determined by trypan blue staining. The cells were diluted in culture medium and plated in 96-well plates at a concentration of 2x10^5^ cells/mL at 0.2 mL per well. After incubating the culture for 24 hours (Jouan incubator - controlled atmosphere 95 % air, 5 % CO_2_, steam saturation, 37 °C), the culture medium was changed to serum-free medium containing the LEGO-LPPOs and incubated for 24 hours in the incubator under the above-mentioned conditions. After 24 h of incubation (37 °C, 5 % CO_2_), cell damage was monitored by determining mitochondrial dehydrogenase activity (MTT assay). The IC_50_ value (mg/L) was calculated from the measured absorbance values at 570 nm^2^. The test was performed in three independent replicates and colistin and polymyxin B were used as positive controls.

Compared to CC50 determined on HepG2 cells, mouse fibroblasts shown higher susceptibility to **LEGO-1-3** (Suppl. Table 1). **LEGO-4** exerts reduced chemical stability caused by the tendency to beta-elimination. Hence, **LEGO-4** is not suitable for practical use. Therefore, the cytotoxicity of **LEGO-4** was not measured.

**Suppl. Table 1.** Cytotoxicity of **LEGO-1-3** toward HepG2 cells (CC50, mg/L) – data previously published in (Do Pham et al., 2022)^1^ and toxicity determined by MTT test in Balb/c 3T3 mouse fibroblast cell line (IC_50_, mg/L).

| Compound | **CC_50_ (mg/L)** | **IC_50_ (mg/L)** |
| --- | --- | --- |
| **LEGO-1** | 33.2 | 12.0 |
| **LEGO-2** | 93.6 | 42.7 |
| **LEGO-3** | 99.4 | 28.5 |

**Minimum inhibitory concentrations of LEGO-LPPOs**

**
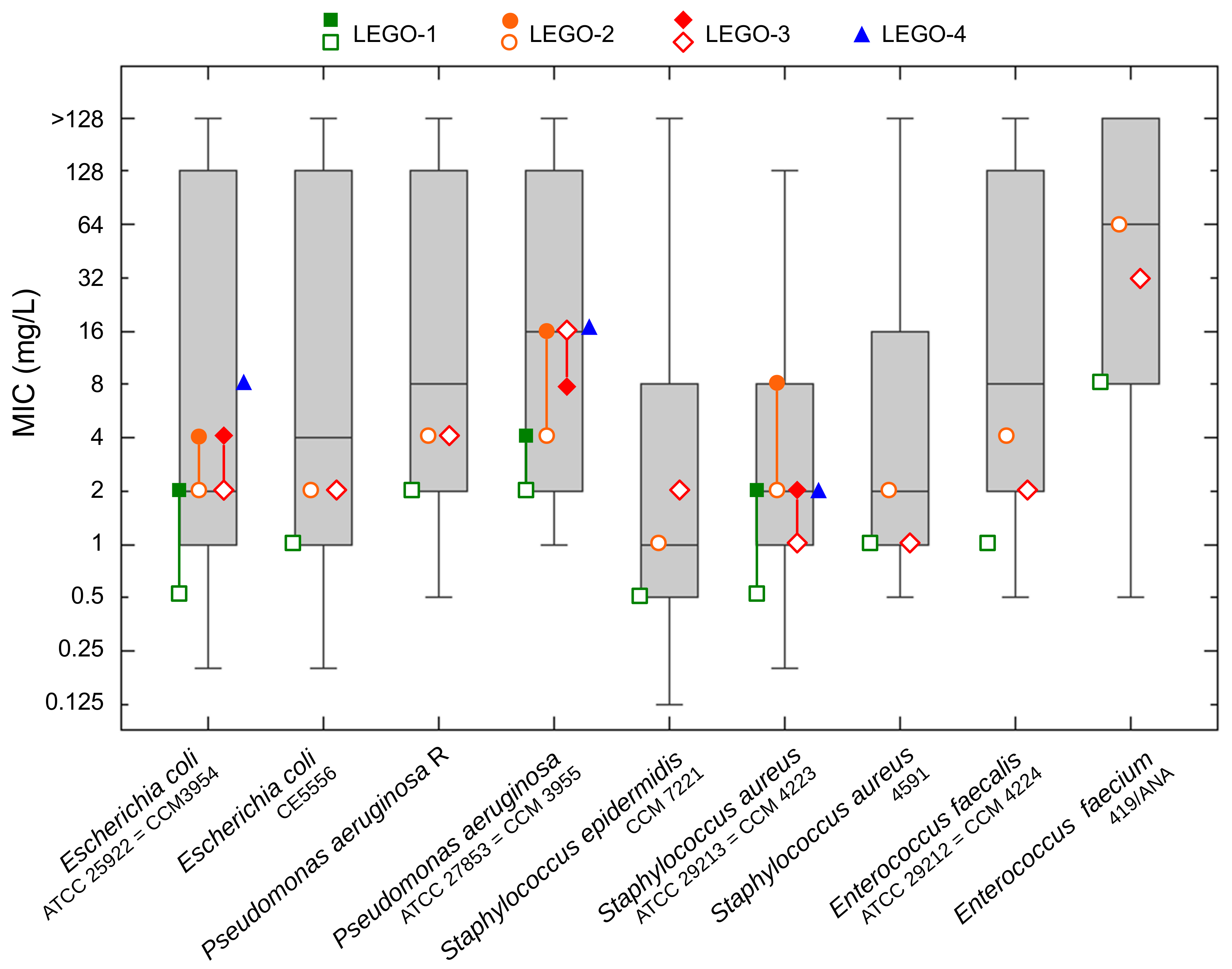
**

**Suppl. Figure 1.** The comparison of antimicrobial activity of all previously published LEGO-LPPOs^1^ against a set of Gram-positive and Gram-negative bacterial strains indicated on *x* axes. The graph shows the distribution of minimal inhibitory concentration (MIC) of the whole set of LEGO-LPPO substances. The comparison of MIC values for LEGO-1-3 published in Do Pham et al., 2022^1^ (open symbols) and LEGO-1-4 in our current study (closed symbols) is indicated. The slight differences in MIC values in the former and in the current study stems from the use of other LEGO-LPPO stock solutions (high concentration in DMSO vs. lower concentration in water).

**Minimal bactericidal concentration and inoculum effect of LEGO-LPPOs**

In order to verify bactericidal activity of LEGO-LPPOs, we determined minimal bactericidal concentration (MBC, Suppl. Table 2) as the lowest concentration that completely inhibited bacterial growth on agar plate after 24 hours of incubation in microtitration plate.

**Suppl. Table 2.** Antimicrobial activity of selected LEGO-LPPOs expressed as minimum bactericidal concentration (MBC, mg/L). MBC was the same (black) or two times higher (red) than MIC. Shown values are average of three repetitions.

| **Compound** | ***E. coli*  CCM 3954** | ***P. aeruginosa* CCM 3955** | ***S. aureus* CCM 4223** | ***E. coli* imp4213** |
| --- | --- | --- | --- | --- |
| **LEGO-1** | N/A | 2 | 1 | N/A |
| **LEGO-2** | N/A | 8 | 2 | N/A |
| **LEGO-3** | N/A | 8 | 2 | N/A |
| **LEGO-4** | 8 | 4 | 2 | 4 |

**
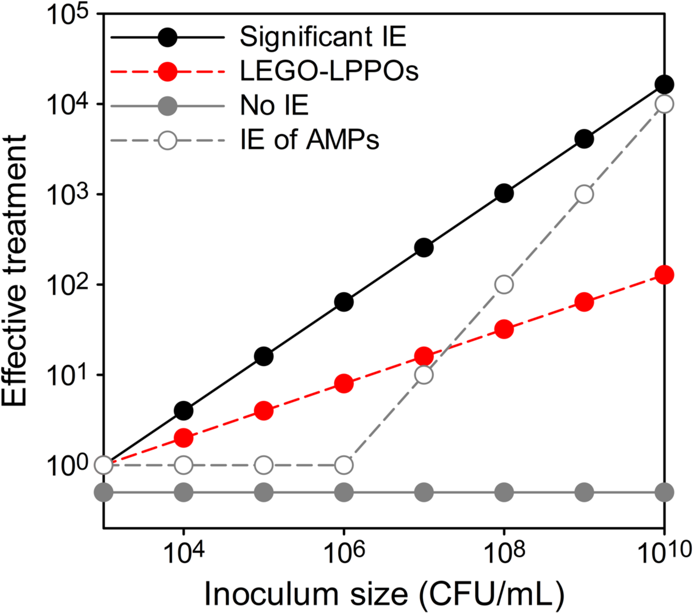
**

**Suppl. Figure 2.** Comparison of observed LEGO-LPPOs inoculum effect (IE, red, dashed line) represented as an average of all **LEGO-4** values on all tested bacterial strains. The significant IE described in literature (black line)^3^ and IE of antimicrobial peptides as previously published (grey dashed line)^4^ is also shown. Theoretical antimicrobial agents with no IE are also presented (grey line). We introduced the parameter of “effective treatment” (*y* axes) for the purpose of comparing different bactericidal agents whose quantities are assessed in different units. The value of effective treatment on represents treatments with bactericidal dosage of model antibacterial agents that is required to kill bacteria to the same extent as the effective concentration of the antibiotic determined at standard conditions.

The MBC values determined after 30 and 120 minutes of incubation of the cells in the presence of **LEGO-4** (Suppl. Fig. 2A and B) were more affected by the IE than the final MIC values. Bactericidal concentration is decreasing through time, as expected. The influence of average IE decreased through time in case of all bacterial strains.

We conclude that the IE – time relations reflect the overall antibiotic susceptibility of each bacterial strain.

**
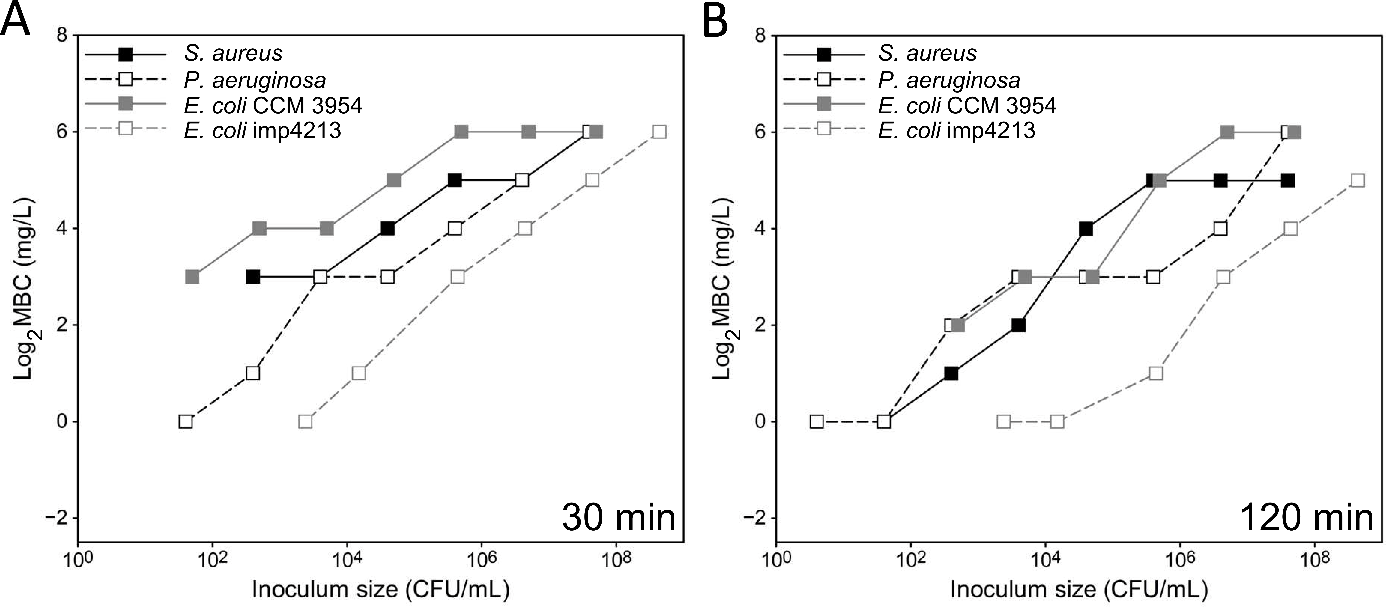
Suppl. Figure 3.** MBC values of **LEGO-4** are affected by IE. MBC was determined in two time points – A) 30 minutes, and B) 120 minutes. Graphs show average of results obtained in two different experiments.

**Mechanism of liposome leakage**

**
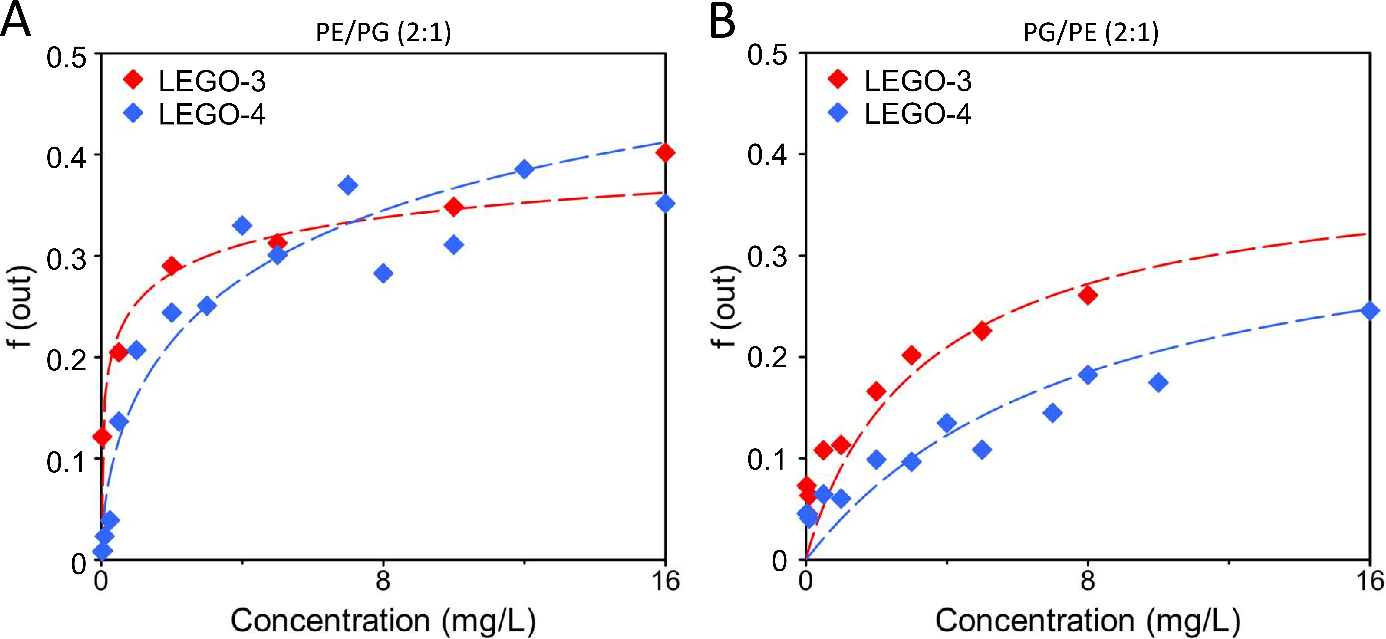
Suppl. Figure 4.** Concentration dependency of LEGO-LPPOs **LEGO-3** and **LEGO-4** activity in ANTS/DPX PE/PG (A) and PG/PE (B) liposomes shown as *f*_out_. The datasets are fitted with Hill function (dashed lines, Eq. 2).

**
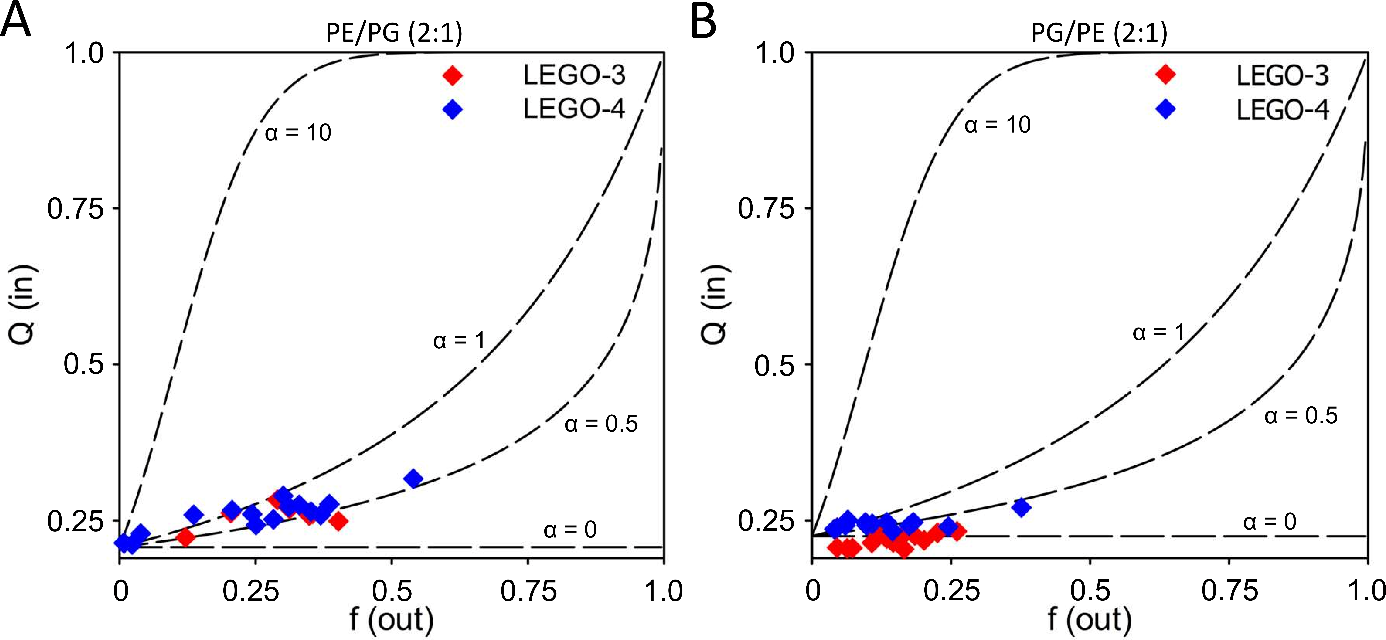
Suppl. Figure 5.** Results of a requenching assay of **LEGO-3** and **LEGO-4** in PE/PG (A) and PG/PE (B) liposomes. Model α values (0, 0.5, 1 and 10) are shown. α ∈ 〈0,5; 1〉 suggest that action mechanism could be graded, slightly anion selective leakage or a combination of “all or none” and graded non-selective leakage.

**Permeabilization of cytoplasmic membrane of living cells**

**
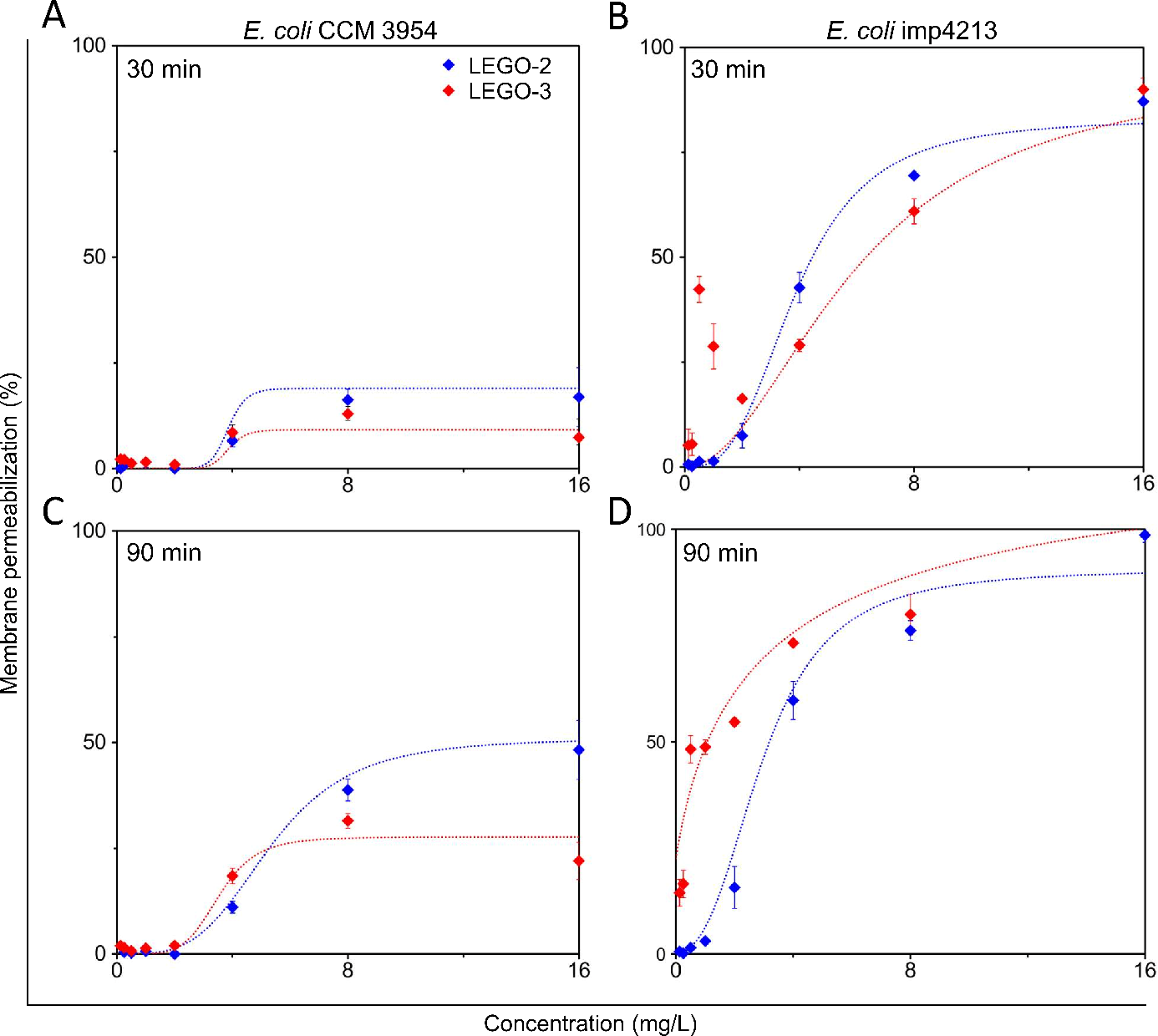
Suppl. Figure 6.** LEGO-LPPOs concentration dependency (**LEGO-2** and **LEGO-3** shown) of *E. coli* CCM 3954 and *E. coli* imp4213 cell permeabilization. The dependency is shown after 30 (A, B) and 90 minutes (C, D) of action. Presented data are averages (±standard deviation) from at least two experiments performed in triplicate. Data were fitted with a Hill function and the parameters are shown in Suppl. Table 3 and 4. Note that in B data obtained for 0.5 and 1 mg/L were omitted from fitting.

**Suppl. Table 3.** Hill function parameters, *E. coli* CCM 3954

| **30 minutes** | | | | |
| --- | --- | --- | --- | --- |
| **Compound** | ***n*** | **k_d_** | **baseline** | ***I*_max_** |
| **LEGO-1** | 3.98 | 2.92 | 0 | 17.57 |
| **LEGO-2** | 22.73 | 3.64 | 1.49 | 7.92 |
| **LEGO-3** | 7.45 | 4.23 | 0 | 16.67 |
| **LEGO-4** | 2.53 | 4.76 | 0.7 | 6 |
| **90 minutes** | | | | |
| **LEGO-1** | 2.96 | 3.96 | 0.1 | 55.84 |
| **LEGO-2** | 5.94 | 3.83 | 1.1 | 27.68 |
| **LEGO-3** | 4.01 | 5.63 | 0.1 | 51.07 |
| **LEGO-4** | 2.37 | 9.55 | 0.1 | 53.61 |

**Suppl. Table 4.** Hill function parameters, *E. coli* imp4213

| **30 minutes** | | | | |
| --- | --- | --- | --- | --- |
| **Compound** | ***n*** | **k_d_** | **baseline** | ***I*_max_** |
| **LEGO-1** | 0.91 | 4.33 | 0.1 | 100 |
| **LEGO-2** | 1.48 | 7.15 | 5 | 100 |
| **LEGO-3** | 3.13 | 3.86 | 0.2 | 82.62 |
| **LEGO-4** | 1.81 | 6.22 | 0.1 | 100 |
| **90 minutes** | | | | |
| **LEGO-1** | 0.83 | 0.95 | 0.1 | 100 |
| **LEGO-2** | 0.92 | 1.23 | 1.9 | 100 |
| **LEGO-3** | 2.83 | 3.22 | 0.5 | 90.57 |
| **LEGO-4** | 0.89 | 1.63 | 0.1 | 100 |

**Affinity of LEGO-LPPOs to bacterial cells**


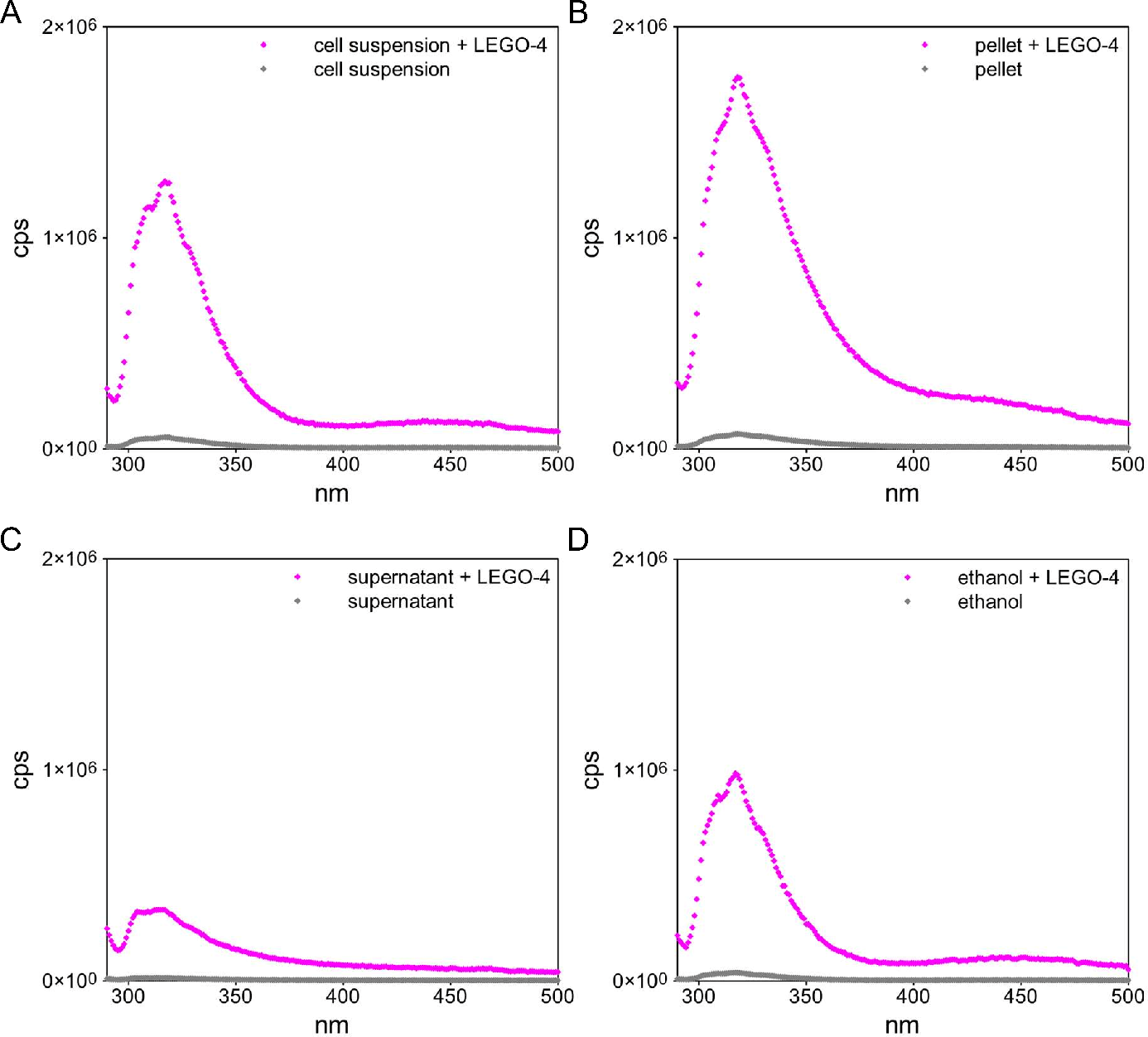


**Suppl. Figure 7.** Fluorescence emission spectra of **LEGO-4** detected in bacterial suspension of *S. aureus* CCM 4223 (OD = 0.2). (A) Original bacterial suspension treated with **LEGO-4** (8 mg/L), (B) pellet and (C) supernatant of this suspension after centrifugation. (D) Control sample of **LEGO-4** in ethanol (8 mg/L). Each graph shows the spectrum of treated and non-treated samples. All samples were diluted in 96% ethanol (84% final concentration) prior to the measurement to avoid different fluorescence of **LEGO-4** in individual environments. Excitation wavelength was 265 nm, see Methods for details.

**Pore forming activity studied by conductivity measurements on planar lipid bilayers**

LEGO-LPPOs kill bacteria by disrupting their cytoplasmic membranes. To compare the pore forming activity of different structures, we measured the conductance on artificial planar membranes of different phospholipid compositions using the method described previously^1^. Experiments were performed on black lipid bilayer membranes of defined phospholipid composition 3% w/v in n-decan/butanol (9:1, v/v). Namely, we used following lipids: 1,2-diphytanoyl-sn-glycero-3-phospho-(1′-rac-glycerol) – DPhPG, 1,2-dioleoyl-sn-glycero-3-phospho-(1′-rac-glycerol) – DOPG and 1,2-dioleoyl-sn-glycero-3-phosphoethanolamine – DOPE, in a ratio 2:1 (m/m) and 1:2 (m/m) (Avanti Polar Lipids, inc., USA) to mimic cytoplasmic membrane of Gram-positive and Gram-negative, respectively. The actual lipid composition is indicated in the respective figure legend. The measurement was performed in the Teflon chamber with *cis* and *trans* compartments connected by the circular aperture (approx. 0.5 mm). Both compartments contained electrolytic buffer (1 M KCl, 10 mM Tris, pH 7.4, filtrated).

Pore-forming activity of **LEGO-1** was previously studied and published^1^. We confirmed and studied the ability of **LEGO-1-4** to make pores in planar membranes of all lipid compositions tested. LEGO-LPPOs were added to the *cis* side of the membrane in concentration from 0.25 to 16 mg/L from stock solution of 10^3^ mg/L. We measured changes in membrane current, which was registered with Ag/AgCl electrodes, amplified by an amplifier (1, 10 or 100 GV/A; MIT) and recorded with a KPCI-3108 card (Keithley). The membrane voltage was 50 mV. Collected data were processed with the QuB software.

We confirmed that compounds **LEGO-1-4** are able to make pores in planar membranes (Supplementary Fig. 7A). Next, we tested the effect of membrane lipid composition on pore-forming activity. We show that **LEGO-4** is making pores of various phenotypes and wide conductance range on both studied membrane models (Supplementary Fig. 7B, C).


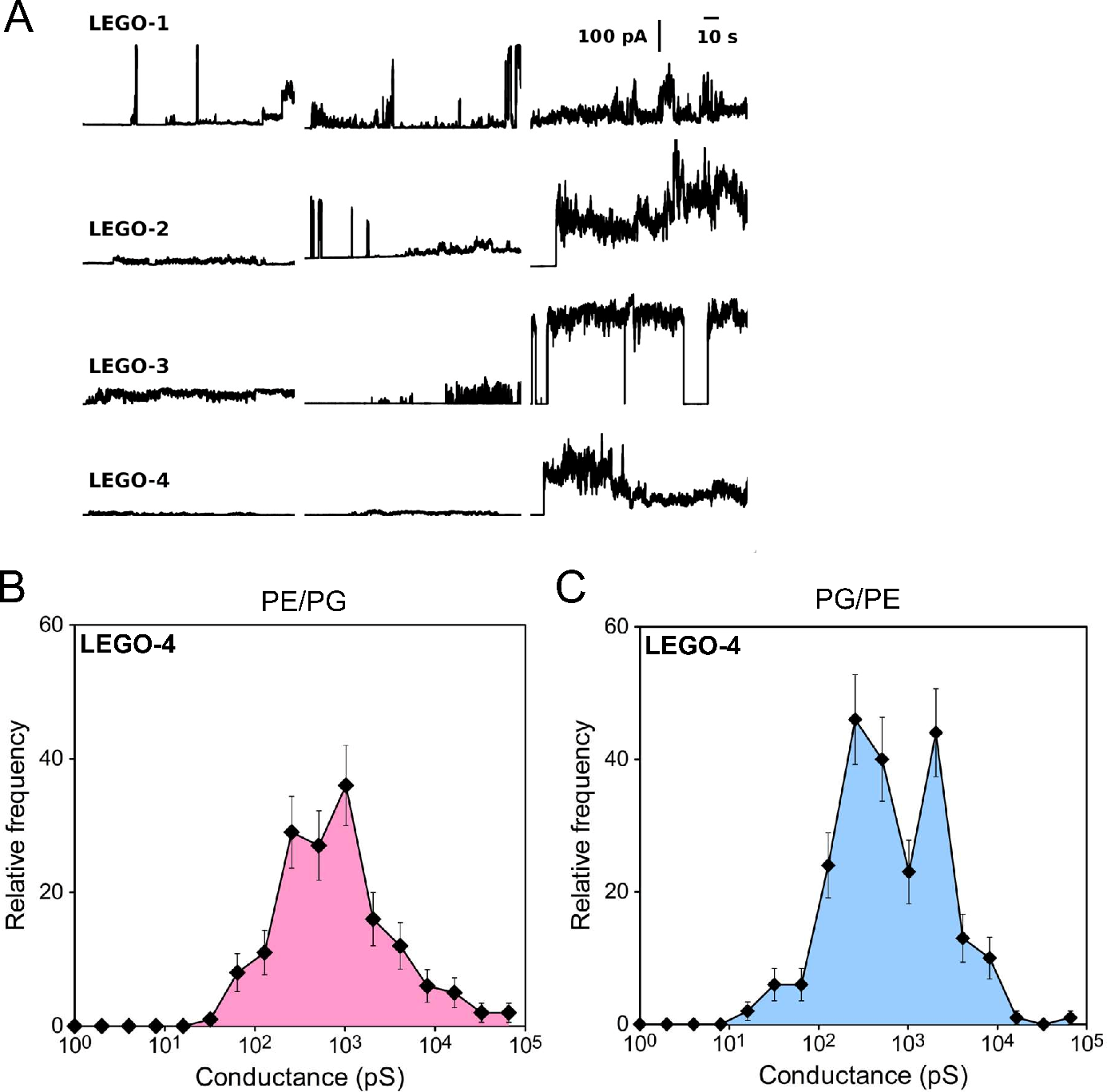


**Suppl. Figure 8.** Pore-forming activity of LEGO-LPPOs. A) Representative examples of single event recordings of conductance measurements induced by LEGO-1-4 in the concentration of 2.5 mg/L over time on planar phospholipid membranes composed of 3% DPhPG (1M KCl, 10mM Tris, pH 7.4, 50 mV applied voltage, 10 GV/A amplifier). Broad distribution of pore sizes can be seen, pores with high noise and the appearance of a step-like increase in conductance were also observed. The noisy character of the recording implicates that the observed events do not correspond to the stable pores with defined stoichiometry. Note that the time resolution of the instrument may not allow to observe the fast pore dynamics (in sub millisecond range). B, C) Histograms of relative frequency of pore conductance (pS) formed by **LEGO-4** in PE/PG 2:1 (A) and PG/PE 2:1 (B) membranes. The ranges of measured conductances were 23 pS to 41 000 pS (PE/PG) and from 12 to 10 000 pS (PG/PE).

**The ability of LEGO-LPPO to disrupt outer membrane of *E. coli* CCM 3954**

We investigated the ability of LEGO-LPPO in the concentration of 2 mg/L to disrupt the outer membrane of *E. coli* CCM 3954 and *E. coli* imp4213 using N-phenyl-1-naphthylamine (NPN) membrane probe fluorescence measurements according to method described previously^5^. Polymyxin B in the concentration of 32 mg/L was used as a positive control.

**
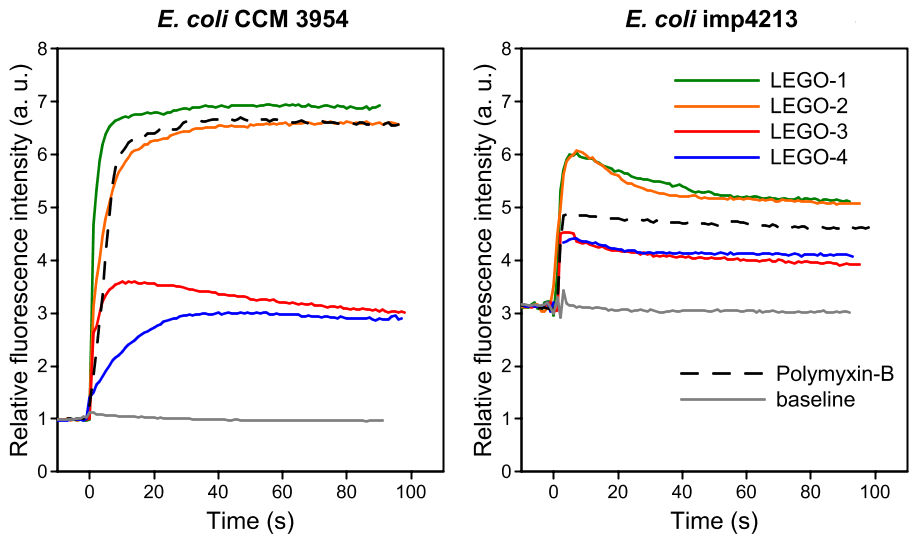
**

**Suppl. Figure 9.** The ability of LEGO-LPPOs in the concentration of 2 mg/L to permeabilize outer membrane of *E. coli* CCM 3954 and *E. coli* imp4213. Polymyxin B was used in the concentration of 32 mg/L. Untreated cells labelled with NPN are shown as the baseline. The graphs show representative curves from two experiments performed in duplicate.

**Composition of systems used in MD simulations and the simulation protocol**

**Suppl. Table 5.** Number of molecules and trajectory length of simulated systems

| **System** | **DOPG** | **DOPE** | **LPPO-1** | **K^+^** | **Cl^-^** | **H_2_O** | **Trajectory length (ns)** |
| --- | --- | --- | --- | --- | --- | --- | --- |
| **PG:PE (2:1)** | 86 | 42 | 0 | 100 | 14 | 6304 | 500 |
| **PG:PE (1:2)** | 42 | 86 | 0 | 56 | 14 | 6304 | 500 |
| **PG:PE (2:1)/LPPO-1** | 86 | 42 | 16 | 100 | 110 | 6304 | 2000 |
| **PG:PE (1:2)/LPPO-1** | 42 | 86 | 16 | 56 | 110 | 6304 | 2000 |

In MD simulations, the leap-frog integrator with 2 fs time step was used. Non-bonded interactions were accounted for using the Verlet cutoff scheme. The neighbor list was updated every 20 steps, with a 1.2 nm cutoff radius for both Coulombic and van der Waals interactions. The Particle-Mesh Ewald method was applied for long-range electrostatic interactions with a real-space cutoff of 1.2 nm. For van der Waals interactions, a force-switch function was employed between 1.0 and 1.2 nm. Temperature of 310 K was maintained using the V-rescale thermostat with a coupling constant of 1.0 ps. Pressure was controlled using the C-rescale barostat with semi-isotropic pressure coupling at 1 bar, a coupling constant of 5 ps, and a compressibility of 4.5×10^-5^ bar^-1^. All bonds were constrained using the LINCS algorithm. Periodic boundary conditions were used with the center-of-mass motion removed every 100 steps.

**References**

1. Do Pham, D. D. *et al.* LEGO-Lipophosphonoxins: A Novel Approach in Designing Membrane Targeting Antimicrobials. *J Med Chem* **65**, 10045–10078 (2022).

2. Sieuwerts, A. M., Klijn, J. G. M., Peters, H. A. & Foekens, J. A. The MTT tetrazolium salt assay scrutinized: how to use this assay reliably to measure metabolic activity of cell cultures in vitro for the assessment of growth characteristics, IC50-values and cell survival. *Eur J Clin Chem Clin Biochem* **33**, 813–824 (1995).

3. Smith, K. P. & Kirby, J. E. The Inoculum Effect in the Era of Multidrug Resistance: Minor Differences in Inoculum Have Dramatic Effect on MIC Determination. *Antimicrob Agents Chemother* **62**, (2018).

4. Bhagunde, P. *et al.* Mathematical modeling to characterize the inoculum effect. *Antimicrob Agents Chemother* **54**, 4739–4743 (2010).

5. Látrová, K. *et al.* Outer membrane and phospholipid composition of the target membrane affect the antimicrobial potential of first- and second-generation lipophosphonoxins. *Scientific Reports 2021 11:1* **11**, 1–16 (2021).
